# Neutrophil-to-Lymphocyte Ratio and Mortality in Cardiovascular Disease or Cancer: A Population-Based Cohort Study

**DOI:** 10.1016/j.jacadv.2025.102362

**Published:** 2025-11-26

**Authors:** Moustafa I. Morsy, Jocelyn M. Friday, Jill P. Pell, Jim Lewsey, Daniel F. Mackay, Ruth Dundas, Tran QB. Tran, Denise Brown, Frederick K. Ho, Claire Hastie, Michael Fleming, Claudia Geue, Alan Stevenson, Clea du Toit, Sandosh Padmanabhan, John G.F. Cleland, Pasquale Maffia, Pierpaolo Pellicori

**Affiliations:** a School of Infection & Immunity, College of Medical, Veterinary and Life Sciences, University of Glasgow, Glasgow, United Kingdom; b School of Health and Wellbeing, College of Medical, Veterinary and Life Sciences, University of Glasgow, Glasgow, United Kingdom; c School of Cardiovascular and Metabolic Health, College of Medical, Veterinary and Life Sciences, University of Glasgow, Glasgow, United Kingdom; d Digital Health Validation Lab, Living Lab, University of Glasgow, United Kingdom; e Department of Pharmacy, School of Medicine and Surgery, University of Naples Federico II, Naples, Italy; f Africa-Europe CoRE in Non-Communicable Diseases & Multimorbidity, African Research Universities Alliance (ARUA) & The Guild of European Research-intensive Universities, Glasgow, United Kingdom

**Keywords:** cardiovascular disease, cancer, epidemiology, heart failure, mortality, neutrophil-to-lymphocyte ratio

## Abstract

**Background:**

Inflammation contributes to development and progression of cardiovascular disease (CVD) and cancer. Whether the neutrophil-to-lymphocyte ratio (NLR), routinely available from blood counts, predicts prognosis in the general population is uncertain.

**Objectives:**

The purpose of this study was to evaluate the association of NLR with mortality in people with and without CVD or cancer.

**Methods:**

In this retrospective cohort study, we used National Health Service records from the Greater Glasgow & Clyde region for people aged >50 years in 2012. Participants were classified hierarchically into 5 exclusive groups: history of cancer, heart failure (HF, or dispensed loop diuretics), CVD, CV risk factors only, or none. Mortality was tracked until December 2019.

**Results:**

We identified 223,388 people with NLR measured between 2014 and 2015: 106,973 (48%) with CV risk factors only, 14,490 (7%) with CVD, 23,009 (10%) with HF or dispensed loop diuretics, 8,677 (4%) with cancer, and 70,239 (31%) none of these features. Median NLR was lowest in the latter group (2.0 [1.5-2.7]) and highest in those with HF or dispensed loop diuretics (2.7 [1.9-3.9]) or cancer (2.7 [1.9-4.1]). Median follow-up was 5.0 years (IQR: 4.4-5.0). In models adjusted for age, sex, estimated glomerular filtration rate, and hemoglobin, the highest NLR quartile predicted higher mortality across all groups (HR [95% CI] 2.07 [1.98-2.17] for CV risk factors and 2.18 [2.02-2.36] for none; 1.87 [1.70-2.04] for CVD, 2.10 [1.98-2.24] for HF or loop diuretics, and 2.30 [2.10-2.52] for cancer).

**Conclusions:**

Higher NLR is associated with greater mortality in adults with and without CVD, HF or cancer, suggesting it could enhance population risk scores.

Cardiovascular disease (CVD) and cancer are common causes of disability, morbidity, and mortality worldwide and share many risk factors.^1^ Smoking, reduced physical activity, and obesity increase the risk of developing both conditions. Cancer and CVD might also share biological mechanisms driving pathophysiology.^2^ Immunoinflammatory responses are involved in the development of atherosclerosis, myocardial infarction, and progression to heart failure (HF),^3^^,^^4^ as well as in different cancer types.^5^ Inflammation-targeted therapies could potentially improve the outcome of many cardiovascular (CV) conditions, including HF^6^, ^7^, ^8^ and inhibit cancer development or facilitate its regression.^9^

The neutrophil-to-lymphocyte ratio (NLR) is a simple, routinely available marker of a disturbed inflammatory state. A higher NLR is associated with worse outcomes in patients with myocardial infarction, diabetes, stroke, HF, and cancer.^10^ However, the generalizability of these findings is limited, as they are usually based on cohorts of highly selected individuals who have been invited and consented to participate in multicenter research registries or trials, or from small, single-center, retrospectively collected samples of patients. Information on general populations is lacking. Therefore, we investigated the distribution and associations of NLR with mortality in middle aged or older adults with or without CV risk factors, overt CVD with or without HF or cancer.

## Methods

### Data source

We used routinely collected National Health Service (NHS) electronic patient records (EPRs) for Greater Glasgow & Clyde from January 1, 2012, to December 31, 2019. NHS Greater Glasgow & Clyde provides health care services to a population of approximately 1.2 million residents across Glasgow City, East Dunbartonshire, East Renfrewshire, Inverclyde, Renfrewshire, and West Dunbartonshire. It is the largest health board in Scotland and among the largest in the United Kingdom. Data were accessed through the NHS West of Scotland Safe-Haven, which is a trusted research environment where pseudonymized health records can be linked for audit and research purposes.^11^ Data comprise demographics, dispensing records from community but not hospital pharmacies, laboratory tests from both primary and secondary care, hospital admissions, including diagnostic and procedural codes, and death records, including certified cause of death. NLR was calculated from lymphocyte and neutrophil counts taken at the same time and date, dividing neutrophil by lymphocyte counts.

### Study population

People aged >50 years on the 1st of January 2012 with an available EPRs were included. Using information acquired from the EPRs during 2012/13, patients were classified into one of 5 mutually exclusive groups in a hierarchical fashion (ie, patients belonging to a group could not belong to any subsequent group) based on hospital records and treatments dispensed in primary care during the 6 months prior to the index date. These 5 groups were: 1) cancer; 2) HF or treatment with a loop diuretic, a surrogate for a missed diagnosis of HF^12^ (HF/LD); 3) established CVD, including ischemic heart disease, myocardial infarction, or stroke; 4) CV risk factors, which included a diagnosis of, or treatment for hypertension, diabetes, or hyperlipidemia; or 5) none of these (without CV risk factors) (Supplemental Figure 1, Supplemental Table 1).

**Figure 1:**
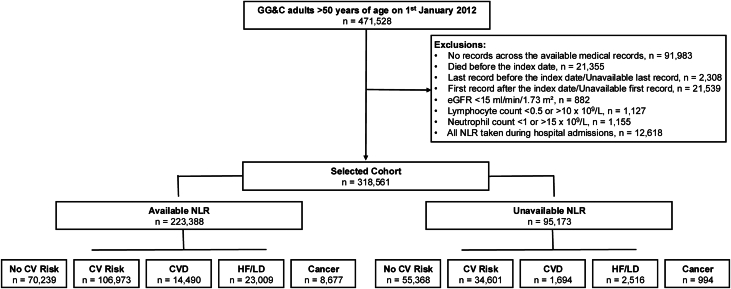
**Flowchart of the Cohort Selection** CV = cardiovascular; CVD = cardiovascular disease; eGFR = estimated glomerular filtration rate; GG&C = Greater Glasgow & Clyde; HF = heart failure; LD = loop diuretic; NLR = neutrophil-to-lymphocyte ratio.

From January 1, 2014, to December 31, 2015, the first available NLR measurement taken in primary care was identified; blood tests taken during hospital admissions were excluded because such results might reflect transient acute illnesses. We also excluded those with end-stage renal disease, defined as an estimated glomerular filtration rate (eGFR) < 15 mL/min/1.73 m^2^, and those with extreme values for lymphocyte (<0.5 or >10 × 10^9^ cells/L) or neutrophil (<1 or >15 × 10^9^ cells/L) counts that might be due to hematological disorders (Figure 1). Furthermore, in a sensitivity analysis, we have excluded patients with a recorded history of malignant hematological cancer (Supplemental Table 2). Patients with a known blood cancer were otherwise included in the cancer cohort.

### Patients characteristics

Comorbidities and procedures were identified through the International Statistical Classification of Diseases and Related Health Problems, 10th Revision codes in any diagnostic or Office of Population Censuses and Surveys Classification of Interventions and Procedures version 4 codes in any procedural position. Diagnostic code lists were derived from CALIBER phenotypes^13^ and existing research literature.^14^^,^^15^ Patients who did not have a diagnostic record for a particular disease were considered to be free of that condition. Baseline treatment and blood results were identified during a 6-month window before the index date. Medications dispensed were identified using the British National Formulary codes (Supplemental Table 3). Data sources and coding systems are described in supplementary materials. The eGFR was calculated using the Chronic Kidney Disease-Epidemiology Collaboration equation without any adjustment for ethnicity.^16^^,^^17^

### Study outcomes

We investigated the association between the first measurement of NLR in 2014/15 and 5-year all-cause mortality and cause of death. Cause of death was identified from National Records Scotland deaths records and classified as CV, cancer, infection, injury, or others (Supplemental Tables 4 and 5). These outcome measures have been assessed in prior studies using data from the same region and data sources.^12^^,^^18^

Individuals were followed up until the first outcome occurred, or until the date of the last available EPRs, whichever came first. The follow-up period concluded on December 31, 2019. To account for potential dropouts due to residential relocation or emigration, the date of the last available EPR was considered the censoring date if it occurred before the end of the follow-up period. Individuals without any available EPRs after the index date were excluded (Figure 1).

### Statistical analysis

Categorical data are presented as numbers and percentages, and continuous data as median and IQR. We used Kaplan-Meier survival analysis and log-rank test to assess the difference in overall survival between diagnostic groups and between quartiles of NLR in each diagnostic group. Mortality rates were calculated as the number of deaths divided by total person-time at risk, expressed per 1,000 person-years. To address the association between NLR and all-cause mortality, we used Cox proportional hazard model to estimate HRs and corresponding 95% CIs, initially adjusting for age and sex, then by adding eGFR and hemoglobin. NLR was modeled as quartiles, taking the first quartile as a reference group, or restricted cubic splines with 4 knots placed at 5th, 35th, 65th, and 95th percentiles, allowing for flexible dynamic of NLR accounting for nonlinearity.^19^ Number of knots was chosen based on Akaike Information Criterion, where the models with the lowest Akaike Information Criterion were considered.^20^ In addition, cross-validation was used to assess model predictive value.^21^ Proportional hazards assumption was checked by visual inspection of scaled Schoenfeld residuals, due to large sample size, and sufficiently met for all the variables. Two-tailed *P* values <0.05 were considered significant. All analyses were conducted with R software version 4.3.0. Packages are listed in supplementary materials.

## Results

**Figure 2:**
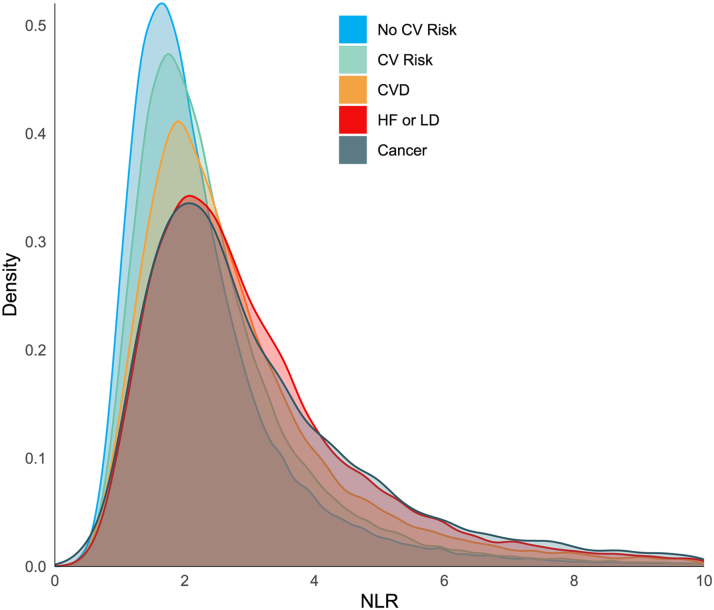
**Density Plot of Baseline Neutrophil-to-Lymphocyte Ratio Distribution per Group** Abbreviations as in Figure 1.

Of 318,561 people, 223,388 (70%) had a valid record of NLR in 2014/15. Of these, prior to 2014, 106,973 (48%) had CV risk factors, 14,490 (7%) had CVD but without HF/LD, 23,009 (10%) had HF/LD, and 8,677 had cancer (4%); 70,239 (31%) had none of the above (Figure 1). Median NLR (IQR) increased with severity of disease, from 2.0 (1.5-2.7) in those free from risk factors or disease to 2.7 (1.9-3.9) in those with HF/LD, and 2.7 (1.9-4.1) in those with cancer (Figure 2, Supplemental Table 6).

Patients with HF/LD (23,009 out of 25,525) and cancer (8,677 out of 9,671) were highly likely to have an NLR available (∼90%); the healthiest cohort (70,239 out of 125,607; 56%) was least likely to have an available measurement. Those in whom NLR was not measured during 2014/15 were generally younger and less likely to have comorbidities; in other words, they had a lower risk profile (Supplemental Table 7).

Tables 1 and 2 illustrate baseline characteristics of each cohort, divided by quartiles of NLR.

**Table 1:** Baseline Demographics, Comorbidities, Blood Tests, and Medications of Cardiovascular Risk Free, Cardiovascular Risk, Cardiovascular Disease, and Heart Failure or Loop Diuretic Cohorts by Quartiles of Neutrophil-to-Lymphocyte Ratio

	No CV Risk				CV Risk				CVD				HF/LD			
	Q1 ≤1.47 (n = 17,677)	Q2 1.48-1.96 (n = 17,552)	Q3 1.97-2.71 (n = 17,546)	Q4 >2.71 (n = 17,464)	Q1 ≤1.61 (n = 27,142)	Q2 1.62-2.16 (n = 26,577)	Q3 2.17-3.00 (n = 27,734)	Q4 >3.00 (n = 25,520)	Q1 ≤1.77 (n =3,657)	Q2 (1.78-2.41) (n =3,602)	Q3 (2.42-3.43) (n =3,618)	Q4 >3.43 (n =3,613)	Q1 ≤1.91 (n =5,758)	Q2 (1.92-2.67) (n =5,766)	Q3 (2.68-3.85) (n =5,758)	Q4 >3.85 (n =5,727)
Age, y	61 (57-67)	61 (57-68)	62 (57-70)	65 (59-74)	67 (60-74)	68 (61-76)	70 (62-78)	73 (65-80)	69 (61-77)	71 (63-79)	74 (66-81)	77 (69-84)	74 (65-81)	76 (68-83)	78 (70-84)	79 (72-86)
Women	11,577 (65%)	10,788 (61%)	9,977 (57%)	9,116 (52%)	16,177 (60%)	14,749 (55%)	14,646 (53%)	13,073 (51%)	1,779 (49%)	1,627 (45%)	1,651 (46%)	1,684 (47%)	3,960 (69%)	3,647 (63%)	3,511 (61%)	3,391 (59%)
DM	Excluded				4,303 (16%)	4,690 (18%)	5,168 (19%)	4,440 (17%)	629 (17%)	667 (19%)	708 (20%)	690 (19%)	1,141 (20%)	1,376 (24%)	1,396 (24%)	1,488 (26%)
IHD					Excluded				2,175 (59%)	2,045 (57%)	1,882 (52%)	1,706 (47%)	1,031 (18%)	1,134 (20%)	1,173 (20%)	1,300 (23%)
MI									993 (27%)	975 (27%)	873 (24%)	739 (20%)	555 (10%)	611 (11%)	610 (11%)	687 (12%)
AF/AFL									850 (23%)	861 (24%)	994 (27%)	1,213 (34%)	636 (11%)	757 (13%)	966 (17%)	1,232 (22%)
PCI									450 (12%)	493 (14%)	364 (10%)	252 (7.0%)	183 (3%)	195 (3%)	173 (3%)	167 (3%)
CABG									78 (2%)	86 (2%)	61 (2%)	59 (2%)	67 (1.2%)	61 (1.1%)	68 (1.2%)	41 (0.7%)
Stroke									786 (21%)	813 (23%)	886 (24%)	955 (26%)	222 (3.9%)	266 (4.6%)	293 (5.1%)	354 (6.2%)
HF									Excluded				788 (14%)	949 (16%)	1,010 (18%)	1,260 (22%)
COPD	234 (1%)	258 (2%)	338 (2%)	656 (4%)	470 (1%)	515 (2%)	689 (3%)	1,028 (4%)	537 (15%)	519 (14%)	597 (17%)	716 (20%)	519 (9.0%)	638 (11%)	760 (13%)	988 (17%)
Blood tests^a^																
Neutrophil count, 10^3^/μL	2.8 (2.3-3.5)	3.6 (3.0-4.3)	4.2 (3.5-5.0)	5.5 (4.4-7.0)	3.2 (2.6-3.9)	4.0 (3.3-4.7)	4.6 (3.8-5.5)	5.8 (4.7-7.3)	3.5 (2.8-4.2)	4.2 (3.5-5.1)	4.8 (4.0-5.8)	6.3 (5.0-7.9)	3.6 (2.9-4.3)	4.4 (3.7-5.3)	5.1 (4.2-6.1)	6.4 (5.1-8.0)
Lymphocyte count, 10^3^/μL	2.5 (2.0-3.0)	2.1 (1.8-2.5)	1.8 (1.5-2.2)	1.4 (1.1-1.8)	2.5 (2.1-3.1)	2.1 (1.8-2.5)	1.8 (1.5-2.2)	1.4 (1.1-1.7)	2.5 (2.1-3.1)	2.0 (1.7-2.5)	1.7 (1.4-2.0)	1.3 (1.0-1.6)	2.5 (2.0-3.0)	1.9 (1.6-2.3)	1.6 (1.3-1.9)	1.1 (0.9-1.4)
eGFR^b^, mL/min/1.73 m^2^	88 (79-95)	88 (78-95)	88 (77-95)	86 (75-95)	83 (72-92)	82 (69-91)	80 (67-90)	77 (61-88)	81 (67-91)	78 (64-90)	77 (61-88)	74 (57-85)	71 (55-84)	67 (50-81)	63 (45-79)	58 (40-76)
CRP, mg/dL	0.3 (0.1-0.4)	0.3 (0.1-0.5)	0.3 (0.2-0.7)	0.5 (0.3-2.0)	0.3 (0.2-0.5)	0.3 (0.2-0.6)	0.3 (0.3-0.8)	0.6 (0.3-2.0)	0.3 (0.2-0.5)	0.3 (0.3-0.8)	0.4 (0.3-1.0)	0.7 (0.3-2.5)	0.4 (0.3-0.8)	0.5 (0.3-1.1)	0.6 (0.3-1.6)	1.0 (0.4-2.9)
Hemoglobin (women), g/dL	13.5 (12.9-14.2)	13.6 (12.9-14.3)	13.6 (12.8-14.3)	13.4 (12.5-14.2)	13.5 (12.7-14.2)	13.4 (12.6-14.2)	13.3 (12.4-14.1)	13.1 (12.0-14.0)	13.1 (12.3-14.0)	13.1 (12.2-14.0)	12.8 (11.9-13.8)	12.7 (11.5-13.7)	13.1 (12.2-14.0)	12.9 (11.9-13.9)	12.7 (11.6-13.7)	12.4 (11.2-13.5)
Hemoglobin (men), g/dL	15.0 (14.2-15.7)	15.0 (14.2-15.7)	15.0 (14.1-15.8)	14.8 (13.7-15.6)	14.8 (13.9-15.6)	14.7 (13.8-15.6)	14.6 (13.6-15.5)	14.3 (13.1-15.3)	14.5 (13.4-15.3)	14.4 (13.4-15.3)	14.2 (12.9-15.2)	13.7 (12.4-14.9)	13.9 (12.7-15.1)	13.9 (12.7-15.0)	13.6 (12.3-14.9)	13.0 (11.7-14.3)
Medications^a^^,^^c^																
Loop diuretics	Excluded				Excluded				Excluded				5,428 (94%)	5,454 (95%)	5,454 (95%)	5,468 (95%)
Thiazides and related					7,144 (26%)	7,299 (27%)	7,754 (28%)	7,245 (28%)	428 (12%)	505 (14%)	524 (14%)	483 (13%)	341 (6%)	313 (5%)	335 (6%)	334 (6%)
ACEI/ARBs					13,230 (49%)	13,518 (51%)	14,421 (52%)	13,018 (51%)	1,851 (51%)	1,861 (52%)	1,852 (51%)	1,575 (44%)	3,240 (56%)	3,484 (60%)	3,358 (58%)	3,177 (55%)
Beta-blocker					8,082 (30%)	8,072 (30%)	8,645 (31%)	7,786 (31%)	1,914 (52%)	1,878 (52%)	1,762 (49%)	1,537 (43%)	2,602 (45%)	2,839 (49%)	2,820 (49%)	2,756 (48%)
MRA					121 (<1%)	125 (<1%)	199 (<1%)	232 (<1%)	32 (<1%)	41 (1%)	32 (<1%)	44 (1%)	399 (6.9%)	431 (7.5%)	491 (8.5%)	591 (10%)
Antiplatelet					9,677 (36%)	10,265 (39%)	11,489 (41%)	11,324 (44%)	2,575 (70%)	2,547 (71%)	2,474 (68%)	2,335 (65%)	2,944 (51%)	3,071 (53%)	3,090 (54%)	3,020 (53%)
Statins	Excluded				16,545 (61%)	16,643 (63%)	17,723 (64%)	15,954 (63%)	2,716 (74%)	2,683 (74%)	2,639 (73%)	2,475 (69%)	3,676 (64%)	3,901 (68%)	3,844 (67%)	3,780 (66%)
Oral anticoagulants					804 (3%)	852 (3%)	1,146 (4%)	1,380 (5%)	501 (14%)	526 (15%)	570 (16%)	595 (16%)	918 (16%)	1,123 (19%)	1,262 (22%)	1,462 (26%)
Insulin					562 (2%)	633 (2%)	796 (3%)	825 (3%)	116 (3%)	113 (3%)	120 (3%)	125 (4%)	253 (4%)	331 (6%)	349 (6%)	419 (7%)
Other hypoglycemics					3,936 (15%)	4,275 (16%)	4,699 (17%)	3,845 (15%)	463 (13%)	485 (13%)	510 (14%)	469 (13%)	929 (16%)	1,131 (20%)	1,108 (19%)	1,106 (19%)
PPI	5,121 (29%)	4,970 (28%)	5,110 (29%)	5,081 (29%)	11,346 (42%)	11,017 (41%)	11,719 (42%)	11,492 (45%)	1,898 (52%)	1,824 (51%)	1,828 (51%)	1,824 (50%)	3,256 (57%)	3,204 (56%)	3,198 (56%)	3,339 (58%)
Corticosteroids (inhaled)	1,313 (7%)	1,525 (9%)	1,689 (10%)	2,141 (12%)	2,606 (10%)	2,822 (11%)	3,255 (12%)	3,847 (15%)	444 (12%)	466 (13%)	521 (14%)	596 (16%)	959 (17%)	1,051 (18%)	1,140 (20%)	1,367 (24%)
Bronchodilators	1,759 (10%)	1,987 (11%)	2,179 (12%)	2,665 (15%)	3,641 (13%)	3,848 (14%)	4,356 (16%)	5,025 (20%)	644 (18%)	636 (18%)	727 (20%)	809 (22%)	1,365 (24%)	1,462 (25%)	1,538 (27%)	1,784 (31%)

Values are median (IQR) or; n (%).

SI conversion factor: To convert C-reactive protein from mg/dL to mg/L, or hemoglobin from g/dL to g/L, multiply by 10.

ACEI = angiotensin-converting enzyme inhibitor; AF = atrial fibrillation; AFL = atrial flutter; ARB = angiotensin receptor blocker; CABG = coronary artery bypass graft; COPD = chronic obstructive pulmonary disease; CRP = C-reactive protein; CV = cardiovascular; CVD = cardiovascular disease; DM = diabetes mellitus; HF = heart failure; IHD = ischemic heart disease; LD = loop diuretic; MI = myocardial infarction; MRA = mineralocorticoid receptor antagonist; PCI = percutaneous coronary intervention; PPI = proton pump inhibitor.

a Most recent blood tests and medication use in the last 6 months before the index date.

b eGFR, estimated glomerular filtration rate using CKD-EPI equation.

c Solely or in combination.

**Table 2:** Baseline Demographics, Comorbidities, Lab Tests, and Medications of Cancer Cohort by Quartiles of Neutrophil-Lymphocyte Ratio

	Q1 ≤1.88 (n = 2,183)	Q2 (1.89-2.67) (n = 2,172)	Q3 (2.68-4.07) (n = 2,157)	Q4 >4.07 (n = 2,165)
Age, y	69 (63-77)	71 (64-78)	73 (65-79)	73 (66-80)
Women	1,158 (53%)	1,072 (49%)	975 (45%)	935 (43%)
DM	247 (11%)	275 (13%)	343 (16%)	319 (15%)
IHD	202 (9%)	206 (10%)	230 (11%)	278 (13%)
MI	83 (4%)	93 (4%)	94 (4%)	138 (6%)
AF/AFL	110 (5%)	111 (5%)	164 (8%)	200 (9%)
PCI	20 (<1%)	32 (2%)	21 (1%)	27 (1%)
CABG	9 (<1%)	<5 (<1%)	5 (<1%)	<5 (<1%)
Stroke	73 (3%)	80 (4%)	83 (4%)	94 (4%)
HF	54 (3%)	56 (3%)	89 (4%)	125 (6%)
COPD	173 (8%)	202 (9%)	249 (12%)	304 (14%)
Blood tests^a^				
Neutrophil count, 10^3^/μL	3.2 (2.5-4.0)	4.1 (3.3-4.9)	4.8 (3.9-6.0)	6.2 (4.9-8.2)
Lymphocyte count, 10^3^/μL	2.2 (1.8-2.8)	1.8 (1.5-2.2)	1.50 (1.2-1.8)	1.0 (0.8-1.3)
eGFR^b^, mL/min/1.73 m^2^	82 (68-91)	81 (66-90)	79 (62-90)	79 (59-91)
CRP, mg/dL	0.4 (0.3-0.9)	0.4 (0.3-1.1)	0.6 (0.3-1.9)	1.4 (0.4-5.1)
Hemoglobin (women), g/dL	13.0 (11.9-13.8)	12.9 (11.9-13.8)	12.7 (11.6-13.6)	12.3 (11.0-13.4)
Hemoglobin (men), g/dL	13.8 (12.6-14.9)	14.0 (12.7-15.0)	13.8 (12.3-14.9)	13.0 (11.5-14.3)
Medications^a^^,^^c^				
Loop diuretics	203 (9%)	236 (11%)	271 (13%)	411 (19%)
Thiazides and related	280 (13%)	313 (14%)	314 (15%)	279 (13%)
ACEI/ARBs	707 (32%)	667 (31%)	731 (34%)	733 (34%)
Beta-blocker	529 (24%)	549 (25%)	520 (24%)	585 (27%)
MRA	16 (<1%)	25 (1%)	34 (2%)	47 (2%)
Antiplatelet	641 (29%)	713 (33%)	744 (34%)	744 (34%)
Statins	835 (38%)	912 (42%)	985 (46%)	965 (45%)
Oral anticoagulants	108 (5%)	111 (5%)	141 (7%)	186 (9%)
Insulin	38 (2%)	45 (2%)	66 (3%)	68 (3%)
Other hypoglycemics	196 (9%)	219 (10%)	264 (12%)	234 (11%)
PPI	1,026 (47%)	992 (46%)	1,063 (49%)	1,216 (56%)
Corticosteroids (inhaled)	203 (9%)	261 (12%)	302 (14%)	353 (16%)
Bronchodilators	321 (15%)	368 (17%)	397 (18%)	490 (23%)

Values are median (IQR) or; n (%) for categorical variables.

SI conversion factor: To convert C-reactive protein from mg/dL to mg/L, or hemoglobin from g/dL to g/L, multiply by 10.

Abbreviations as in Table 1.

a Most recent blood tests and medication use in the last 6 months before the index date.

b eGFR, estimated glomerular filtration rate using CKD-EPI equation.

c Solely or in combination.

Across all CV diagnostic and cancer groups, those in the highest quartile of NLR were more likely to be older and to be men and to have lower hemoglobin and eGFR than those in the lowest quartile. There was also a substantial increase in C-reactive protein (CRP) levels among those in the highest NLR quartile. However, CRP was missing for >60% of the population (Supplemental Table 6).

**Figure 3:**
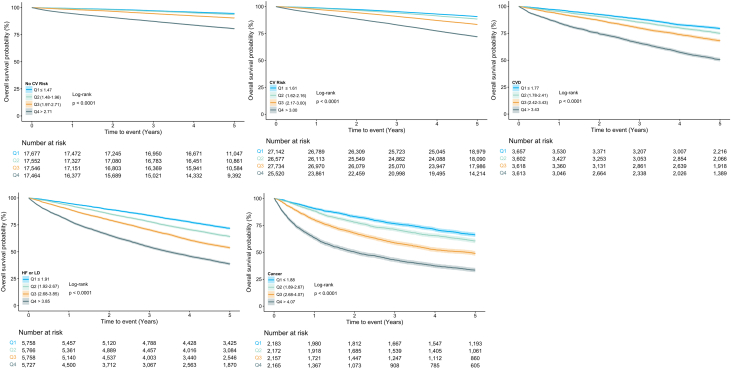
**Kaplan-Meier Estimates of 5-Year Survival by Quartiles of Neutrophil-to-Lymphocyte Ratio** Abbreviations as in Figure 1.

**Figure 4:**
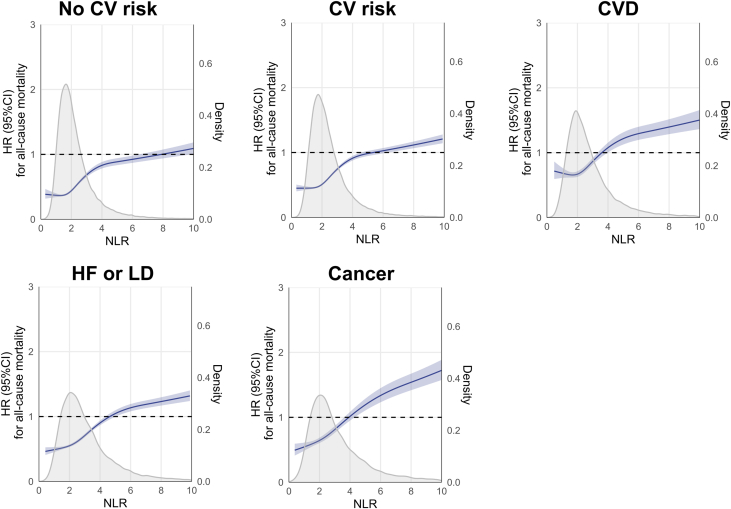
**Association Between Baseline Neutrophil-to-Lymphocyte Ratio and 5-Year All-Cause Mortality** Shading indicates 95% CI; and solid lines, HRs adjusted for age, sex, eGFR, and hemoglobin. Density plot showing the distribution of NLR in each group. Survival associations were assessed with restricted cubic splines with knots at 5th, 35th, 65th, and 95th percentiles of neutrophil-lymphocyte ratio distribution. Abbreviations as in Figure 1.

During a median follow-up of 5.0 years IQR (4.4-5.0), 42,099 (19%) people died. Compared to others, patients with cancer had the highest all-cause mortality, followed by those with HF/LD (Supplemental Figure 2). For all cohorts (Figure 3), patients in the highest quartile of NLR had the highest probability of mortality. In full adjusted Cox proportional hazard models, NLR was associated with a higher mortality across all groups (Figure 4, Table 3).

**Table 3:** Associations With 5-Year All-Cause Mortality in Patients With or Without Cardiovascular Risk Factors/Disease or Cancer by Quartiles of NLR at Baseline

Group, NLR quartile	Model 1			Model 2		
	No. of Events/No. of Patients	Rate per 1,000 Person-Years (95% CI)	HR (95% CI)	No. of Events/No. of Patients	Rate per 1,000 Person-Years (95% CI)	HR (95% CI)
No CV risk						
Q1 ≤1.47	910/17,677	10.97 (10.27-11.71)	Reference	826/15,664	11.25 (10.50-12.04)	Reference
Q2 (1.48-1.96)	1,077/17,552	13.12 (12.35-13.92)	1.10 (1.01-1.20)	960/15,558	13.2 (12.37-14.06)	1.10 (1.00-1.21)
Q3 (1.97-2.71)	1,620/17,546	20.12 (19.15-21.12)	1.47 (1.35-1.59)	1,446/15,691	20.08 (19.05-21.14)	1.41 (1.29-1.54)
Q4 >2.71	3,281/17,464	43.77 (42.29-45.30)	2.40 (2.22-2.58)	2,974/15,736	44.09 (42.52-45.70)	2.18 (2.02-2.36)
CV risk						
Q1 ≤1.61	2,453/27,142	19.26 (18.51-20.04)	Reference	2,351/25,863	19.38 (18.60-20.18)	Reference
Q2 (1.62-2.16)	2,993/26,577	24.26 (23.40-25.15)	1.13 (1.07-1.19)	2,865/25,413	24.29 (23.41-25.19)	1.12 (1.06-1.18)
Q3 (2.17-3.00)	4,503/27,734	35.93 (34.89-36.99)	1.47 (1.40-1.54)	4,306/26,620	35.77 (34.71-36.85)	1.42 (1.35-1.49)
Q4 >3.00	6,996/25,520	65.46 (63.94-67.02)	2.23 (2.13-2.33)	6,694/24,509	65.15 (63.6-66.73)	2.07 (1.98-2.17)
CVD						
Q1 ≤1.77	734/3,657	45.57 (42.33-48.99)	Reference	692/3,444	45.63 (42.29-49.16)	Reference
Q2 (1.78-2.41)	880/3,602	56.97 (53.27-60.86)	1.12 (1.01-1.23)	829/3,411	56.57 (52.79-60.56)	1.11 (1.01-1.23)
Q3 (2.42-3.43)	1,127/3,618	76.24 (71.86-80.83)	1.29 (1.18-1.42)	1,053/3,408	75.42 (70.93-80.12)	1.26 (1.14-1.39)
Q4 >3.43	1,747/3,613	138.92 (132.48-145.59)	2.02 (1.85-2.20)	1,625/3,396	136.64 (130.07-143.44)	1.87 (1.70-2.04)
HF/LD						
Q1 ≤1.91	1,595/5,758	65.24 (62.08-68.52)	Reference	1,532/5,550	64.96 (61.75-68.29)	Reference
Q2 (1.92-2.67)	2,034/5,766	87.66 (83.89-91.55)	1.17 (1.10-1.25)	1,964/5,565	87.65 (83.82-91.61)	1.15 (1.08-1.23)
Q3 (2.68-3.85)	2,617/5,758	122.94 (118.27-127.74)	1.53 (1.44-1.63)	2,531/5,574	122.75 (118.01-127.63)	1.47 (1.38-1.57)
Q4 >3.85	3,451/5,727	196.65 (190.15-203.33)	2.30 (2.17-2.44)	3,316/5,523	195.36 (188.76-202.12)	2.10 (1.98-2.24)
Cancer						
Q1 ≤1.88	728/2,183	83.44 (77.49-89.73)	Reference	701/2,083	84.42 (78.29-90.91)	Reference
Q2 (1.89-2.67)	846/2,172	103.68 (96.81-110.91)	1.17 (1.06-1.30)	819/2,091	104.34 (97.31-111.73)	1.20 (1.08-1.32)
Q3 (2.68-4.07)	1,087/2,157	154.46 (145.41-163.92)	1.65 (1.50-1.81)	1,037/2,065	153.66 (144.45-163.30)	1.59 (1.44-1.75)
Q4 >4.07	1,420/2,165	262.12 (248.66-276.11)	2.61 (2.39-2.86)	1,373/2,082	264.92 (251.09-279.31)	2.30 (2.10-2.52)

NLR = neutrophil-to-lymphocyte ratio; other abbreviations as in Table 1.

Model 1: adjusted for age and sex.

Model 2: adjusted for age, sex, eGFR, and hemoglobin.

In a sensitivity analysis that excluded 799 people (9% of the total cancer population) with a history of hematological malignancies, findings did not substantially change: compared to the lowest quartile, patients with cancer in the highest NLR quartile had a greater risk of death (HR [95% CI]; 2.46 [2.23-2.71], when the model was adjusted for age, sex, eGFR, and hemoglobin) (Supplemental Figure 3).

Patients with HF or CVD were more likely to die of CV causes compared to other diagnostic groups. For patients without cancer, those in the highest quartile of NLR had more deaths due to infection, and less due to cancer, compared to the lowest quartile. Most patients with cancer on the index date died of cancer, particularly those in the highest quartile of NLR (Supplemental Figure 4).

## Discussion

This analysis shows that NLR, a routinely measured, readily available, blood marker that can easily be obtained from EPRs (Central Illustration), might be used to stratify mortality risk for a broad range of patients at risk or with CVD or cancer.

**Central Illustration:**
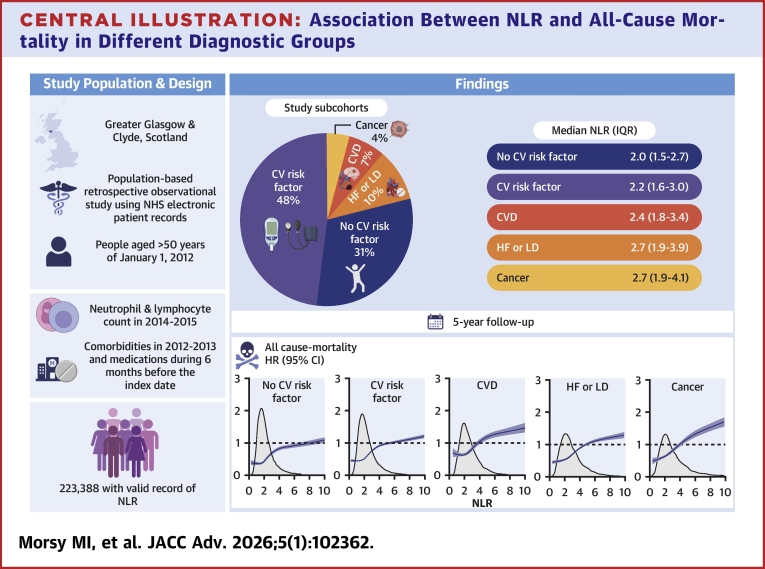
**Association Between NLR and All-Cause Mortality in Different Diagnostic Groups** Created in BioRender. Maffia, P. (2025, https://BioRender.com/ucz89qr). NHS = National Health Service; other abbreviations as in Figure 1.

Older age was associated with higher values of NLR. This might just reflect a higher burden of comorbidities and disease in older people. Age might depress neutrophil function, but it has little effect on their numbers.^22^^,^^23^ However, aging is associated with a decline in circulating lymphocytes,^24^^,^^25^ and consequently an increase in NLR. Thus, a higher NLR might reflect both a greater disease burden and the effects of age on the immune system. As expected, we found that a higher NLR was directly associated with another marker of inflammation, CRP. Heightened inflammation may cause coronary and systemic vascular damage,^9^ impair cardiac function, or promote tumor development and progression leading to worse clinical outcomes.^26^^,^^27^ Anti-inflammatory therapy with canakinumab, or colchicine but not methotrexate has been successful in reducing the risk of CV events in individuals with a previous myocardial infarction, associated with reductions in CRP.^6^^,^^28^, ^29^, ^30^, ^31^ Canakinumab reduced NLR but methotrexate lowered the lymphocyte count and therefore increased NLR.^32^ Finally, modulation of the immune system through immune checkpoint inhibitors has modified the history of disease of many types of cancers.^33^ NLR has been suggested as a biomarker for tumor response to immune checkpoint inhibitors, with decrease in NLR after therapy associated with better patient outcomes.^34^^,^^35^

Our findings agree with, and expand on, those from other large-scale population studies, suggesting that higher NLR predicts a higher mortality.^36^^,^^37^ Interestingly, the association between NLR and mortality persists even among individuals apparently free of risk factors for CVD. For this group, those in the highest quartile of NLR had a 1-year mortality of almost 10%, suggesting the presence of undiagnosed severe disease. These findings support those from a population-based, prospective cohort study in an older community-dwelling population from the Netherlands (n = 8,715), of whom <10% had a history of CVD or cancer. In that cohort (mean age 66 years), participants in the highest quartile of NLR had a greater risk of death compared to the lowest quartile.^38^ In another study that combined 2 Dutch cohorts (n = 14,433), of younger (mean age 47.3 years) and healthier people, NLR did not associate with all-cause mortality.^39^

Overall, our findings suggest that the integration of NLR into routine clinical practice could enhance the early identification of high-risk patients with or at risk of a range of different diseases in primary and secondary care. One of the strengths of our study is the use of large, routinely collected population-level data, which enhances the generalizability of our findings.

However, further research is warranted to elucidate the underlying mechanisms of these associations, and to refine the clinical applications of NLR in various clinical contexts.

### Study limitations

The observational nature of this study precludes causal inferences. Unlike registries and clinical trials, our data were derived from EPRs of people who had some reason for having a blood test. Additionally, missing or inaccurately recorded information in the EPRs could lead to misclassification of patient characteristics or outcomes, potentially biasing our results. Although we excluded blood tests performed during hospitalizations and those that suggested possible undiagnosed hematological cancers, other potential confounders such as concurrent infections, medications, and/or other undiagnosed or diagnosed conditions requiring long-term monitoring might have influenced NLR and affected our results. Importantly, smoking, blood pressure and body mass index might influence NLR and prognosis,^40^ but this information is not captured in the EPRs to which we had access. Additionally, NLR was measured at a single time point; longitudinal changes in NLR were not assessed.

## Conclusions

NLR is a simple, widely available blood marker. Higher values of NLR are associated with greater age, higher blood CRP, a higher prevalence of CV and non-CV comorbidities and cancer, and a greater risk of death. Further studies should be done to elucidate the mechanisms linking NLR with adverse outcomes, to determine the value of adding it to CV risk scores, and to explore its potential as a therapeutic target.

## Funding support and author disclosures

The oGRE Challenge is supported by the Glasgow Living Laboratory for Precision Medicine funded by the UKRI Strength in Places Fund (SIPF00007/1). Data extraction and record linkage was performed by the West of Scotland Safe Haven service (IRAS Project ID 321198) at NHS Greater Glasgow and Clyde, under local ethical approval GSH22ME007. Dr Maffia is supported by 10.13039/501100000274British Heart Foundation grants (PG/19/84/34771, FS/19/56/34893A, PG/21/10541, PG/21/10634, PG/23/11680, and PG/24/11946), the Italian Ministry of University and Research (MUR) PRIN 2022 (2022T45AXH), funded by the European Union-Next Generation EU, Mission 4, Component 1, CUP E53D23012760006, and the European Union-Next Generation EU, Project CN00000041, Mission 4, Component 2, CUP B93D21010860004. Drs Maffia, Pellicori, and Cleland are supported by the Heart Research UK grant (SCOT24-100004). Drs Friday and Cleland are supported by the 10.13039/501100000274British Heart Foundation Centre for Research Excellence Grant (grant number RE/18/6/34217). Dr Morsy is supported by the RS Macdonald Charitable Trust “Seedcorn Funding for Multidisciplinary Stroke Research” (Grant GA-03503). Dr Pellicori has received consultancy honoraria and/or sponsorship support from Boehringer Ingelheim, Pharmacosmos, Novartis, Vifor, AstraZeneca, and Caption Health and research support from Bristol Myers Squibb in the past 5 years, not connected with this manuscript. Dr Cleland has received grants from British Heart Foundation, Pharma Nord, and Pharmacosmos; personal fees from Abbott, Amgen, Novartis, Medtronic, Idorsia, Servier, AstraZeneca, Innolife, Torrent, and Respicardia; grants and personal fees from Bayer, Bristol Myers Squibb, Vifor, Johnson & Johnson, Myokardia, and Viscardia; and personal fees and nonfinancial support from Boehringer Ingelheim and NI Medical. All other authors have reported that they have no relationships relevant to the contents of this paper to disclose.
