# Supplementary material for: Neutrophil-to-Lymphocyte Ratio and Mortality in Cardiovascular Disease or Cancer: A Population-Based Cohort Study
Source: JACC Adv. 2025 Nov 26;5(1):102362. doi: 10.1016/j.jacadv.2025.102362 (PMC12702164; doi:10.1016/j.jacadv.2025.102362)
Supplement: Supplemental Material [file mmc1.docx]

Table of Contents

[SMR01 2](#_Toc205384670)

[SMR04 2](#_Toc205384671)

[PIS 2](#_Toc205384672)

[Coding Systems 2](#_Toc205384673)

[Supplemental Table 1: Codes used to categorize cohorts and corresponding databases. Unless stated otherwise, all nested codes were included. 3](#_Toc205384674)

[Supplemental Table 2: Codes used to identify hematological malignancies. 9](#_Toc205384675)

[Supplemental Table 3: BNF codes used to identify dispensed medications at baseline. Unless stated otherwise, all nested codes were included. 9](#_Toc205384676)

[Supplemental Table 4: Codes used to identify cause of death. 11](#_Toc205384677)

[Supplemental Table 5: Codes used to identify death due to infection. Unless stated otherwise, all nested codes were included. 12](#_Toc205384678)

[Supplemental Figure 1: Flow diagram of categorization of study individuals based on prevalent disease and dispensed medications. CV, Cardiovascular; CVD, Cardiovascular disease; DM, Diabetes mellitus; GG&C; Greater Glasgow & Clyde, HF, Heart failure; LD, Loop diuretic. 13](#_Toc205384679)

[Supplemental Table 6: Baseline demographics, comorbidities, blood tests and medications of the overall population and cardiovascular risk free, cardiovascular risk, cardiovascular disease, heart failure or loop diuretic and cancer cohorts with available NLR during 2014-2015. 14](#_Toc205384680)

[Supplemental Table 7: Baseline demographics, comorbidities, blood tests and medications of the overall population and cardiovascular risk free, cardiovascular risk, cardiovascular disease, heart failure or loop diuretic and cancer cohorts with no available NLR during 2014-2015. 16](#_Toc205384681)

[Supplemental Figure 2: Kaplan-Meier estimates of 5-year survival between groups by prevalent cardiovascular risk factors/disease or cancer. CV, Cardiovascular; CVD, Cardiovascular disease; HF, Heart failure; LD, Loop diuretic 17](#_Toc205384682)

[Supplemental Figure 3: 5-year survival analysis in people with prevalent cancer excluding those with records of hemotalogical cancer. (A) Kaplan-Meier estimates by quartiles of NLR. (B) Association between NLR and all-cause mortality, adjusting for age, sex, eGFR and hemoglobin. (C) Association between NLR quartiles and all-cause mortality taking the 1^st^ quartile as reference. Model 1 adjusted for age and sex. Model 2 adjusted for age, sex, eGFR and hemoglobin. 18](#_Toc205384683)

[Supplemental Figure 4: Cause specific mortality by quartiles of baseline neutrophil-to-lymphocyte ratio (NLR) in patients with or without cardiovascular risk factors/disease or cancer. CV, Cardiovascular; CVD, Cardiovascular disease; HF, Heart failure; LD, Loop diuretic. 19](#_Toc205384684)

[References 20](#_Toc205384685)

**Data Sources**

## **SMR01**

The Scottish Morbidity Records 1 (SMR01) is the largest database in the SMR series, containing episode-level data for all general/acute inpatient or day cases in Scottish NHS hospitals or Scottish NHS beds in non-NHS institutions. An SMR01 record is generated for an inpatient or day case when they are admitted to an NHS hospital from an external location; when they are admitted to a contracted NHS bed in a non-NHS institution; when they change specialty; when they transfer from another NHS hospital; or when they change consultant but not specialty. Additionally, an SMR01 record is created when an inpatient moves into and/or out of a facility or is readmitted after being discharged for more than five days.

## **SMR04**

The Scottish Morbidity Records 4 (SMR04), captures data on psychiatric inpatient and day case episodes in Scottish NHS hospitals, including patient demographics, admission details, psychiatric diagnoses, procedures, and legal status under mental health legislation.

## **PIS**

The Prescription Information System (PIS) in Scotland captures detailed data on all NHS prescriptions dispensed in the community (1). It includes, medication details, and prescribing and dispensing records.

# **Coding Systems**

International Classification of Diseases, 10^th^ Revision (ICD-10) is a global standard for coding diseases, conditions, and health-related problems provided by the World Health Organization (WHO). It categorizes medical diagnoses and procedures into alphanumeric codes, where each code represents a specific disease or condition with a hierarchical structure to allow for detailed classification.

Office of Population Censuses and Surveys Classification of Interventions and Procedures, Version 4 (OCSP-4) is another hierarchical alphanumeric coding system used in the UK governed by Crown Copyright to classify surgical procedures and interventions.

The British National Formulary (BNF) is a pharmaceutical reference book used in the UK, published jointly by the British Medical Association and Royal Pharmaceutical Society. It lists medicines available on the NHS, detailing their indications, dosages, side effects, and contraindications. The BNF categorizes medications by therapeutic class and provides guidance on prescribing and administration to healthcare professionals. The NHS Business Service Authorly assigns a unique, hieratical code to all included medications and chemicals, and this code is referred to as the BNF code.

**Supplemental Table 1: Codes used to categorize cohorts and corresponding databases.** Unless stated otherwise, all nested codes were included.

| **Data Source** | **Code Source** | **Code** | **Description** |
| --- | --- | --- | --- |
| **CV Risk** | | | |
| SMR01 & SMR04 | ICD-10  ICD-10  ICD-10  ICD-10  ICD-10  ICD-10  ICD-10  ICD-10  ICD-10  ICD-10  ICD-10  ICD-10  ICD-10  ICD-10  ICD-10  ICD-10  ICD-10 | E10  E11  E12  E13  E14  G59.0  G63.2  H28.0  H36.0  M14.2  N08.3  O24.1  O24.2  O24.3  Q24.0  Q24.1  Q24.3 | DM  (CALIBER Phenotype) |
| SMR01 & SMR04 | OPCS-4  OPCS-4  ICD-10  ICD-10  ICD-10  ICD-10  ICD-10 | X82.8  X82.9  I10  I11  I12  I13  I15 | Hypertension  (CALIBER Phenotype) |
| PIS | BNF | 0205051 | Angiotensin converting enzyme |
| PIS | BNF | 0205052  0206020Z0 | Angiotensin receptor blockers  Valsartan/Amlodipine |
| PIS | BNF | 0205052AE | Sacubitril/Valsartan |
| PIS | BNF | 0209 | Antiplatelet |
| PIS | BNF | 020502 | Centrally-acting antihypertensive |
| PIS | BNF | 0204 | Beta-adrenergic blocking agents |
| PIS | BNF | 020602  0205052AB  0205052AC  0206020C0  0206020T0 | Calcium-channel blockers  Olmesartan medoxomil/amlodipine  Olmesartan medoxomil/amlodipine/hydrochlorothiazide  Diltiazem hydrochloride  Verapamil hydrochloride |
| PIS | BNF | 0201010F | Digoxin |
| PIS | BNF | 0209000A0  0204000AC  0209000V0 | Aspirin  Bisoprolol fumarate/aspirin  Dipyridamole and Aspirin |
| PIS | BNF | 0202030X0  0202030S0  0202040G0  0202040T0  0202040S0 | Eplerenone  Spironolactone  Co-flumactone(Hydroflumethiazide/spironolactone)  Spironolactone with loop diuretics  Spironolactone with thiazides |
| PIS | BNF | 020802 | Oral anticoagulants |
| PIS | BNF | 020201  0202040H  0202040C0  0204000Y0  020400040  0205051AB  0202040A0  0202040S0  0202040V0  0202080B0  0205052Y0  020400010  020400030  0204000F0  0204000Q0  0204000W0  0205051H0  0205051K0  0205051N0  0205051P0  0205051Z0  0205052A0  0205052P0  0205052R0  0205052X0  0205052AC  0205051G0 | Thiazides and related  Co-triamterzide (Triamterene/hydrochlorothiazide)  Co-amilozide(Amiloride hydrochloride/hydrochlorothiazide)  Co-prenozide (Oxprenolol hydrochloride/ cyclopenthiazide)  Co-tenidone (Atenolol/chlortalidone)  Perindopril tosilate/indapamide  Amiloride hydrochloride with thiazides  Spironolactone with thiazides  Triamterene with thiazides  Bendroflumethiazide/potassium  Olmesartan medoxomil/hydrochlorothiazide  Pindolol with diuretic  Timolol with diuretic  Atenolol with diuretic  Propranolol hydrochloride with diuretic  Metoprolol tartrate with diuretic  Enalapril maleate with diuretic  Lisinopril with diuretic  Perindopril erbumine with diuretic  Quinapril hydrochloride with diuretic  Perindopril arginine with diuretic  Irbesartan with diuretic  Losartan potassium with diuretic  Telmisartan with diuretic  Valsartan with diuretic  Olmesartan medoxomil/amlodipine/ hydrochlorothiazide  Co-zidocapt (Hydrochlorothiazide/captopril) |
| PIS | BNF | 0601023AU  060101 | Insulin/liraglutide  Insulin |
| PIS | BNF | 0601023AU  0601023S0  0601023V0  0601023B0  0601023W0  0601023S0  0601023V0  0601023B0  0601023W0  0601022B0  0601023AD  0601023AF  0601023AH  0601023AJ  0601023AL  0601023AP  0601023AR  0601023V0  0601023W0  0601023Z0  0601023AB  0601023AS  0601023Y0  0601023AI  0601023AQ  0601023AK  0601021A0  0601021M0  0601021X0  0601021P0  0601023AE  0601023AA | Insulin/liraglutide  Rosiglitazone  Metformin hydrochloride/ Rosiglitazone  Pioglitazone hydrochloride  Metformin hydrochloride/ Pioglitazone  Rosiglitazone  Metformin hydrochloride/ Rosiglitazone  Pioglitazone hydrochloride  Metformin hydrochloride/ Pioglitazone  Metformin  Metformin/sitagliptin  Linagliptin/metformin  Saxagliptin/metformin  Alogliptin/metformin  Dapagliflozin/metformin  Canagliflozin/metformin  Empagliflozin/metformin  Metformin/rosiglitasone  Metformin/pioglitasone  Metformin/vildagliptin  Liraglutide  Albiglutide  Exenatide  Lixisenatide  Dulaglitide  Alogliptin  Glimepiride  Glicazide  Tolbutamide  Glipizide  linagliptin  vildagliptin |
| PIS | BNF | 0212000B0  0212000AJ  0212000M0  0212000X0  0212000AA  0212000Y0  0212000AC | Atorvastatin  Fenofibrate/simvastatin  Fluvastatin sodium  Pravastatin sodium  Rosuvastatin calcium  Simvastatin  Simvastatin/ezetimibe |
| **CV Disease** | | | |
| SMR01 & SMR04 | ICD-10  ICD-10  ICD-10  ICD-10  ICD-10  ICD-10 | I71.3  I71.4  I71.5  I71.6  I71.8  I71.9 | AAA  (CALIBER Phenotype) |
| SMR01 & SMR04 | ICD-10  ICD-10  ICD-10  OPCS-4  OPCS-4  ICD-10  ICD-10 | I25.2  I23  I24.1  K50.2  K50.3  I21  I22 | MI  At least on record of  (acute myocardial infarction, complications of myocardial infarction, subsequent myocardial infarction, or not otherwise specified myocardial infarction) based on CALIBER phenotypes |
| SMR01 & SMR04 | ICD-10 | I48 | AF/AFL  (CALIBER phenotype for definite diagnosis) |
| SMR01 & SMR04 | OPCS-4  OPCS-4  OPCS-4  OPCS-4  OPCS-4  OPCS-4  OPCS-4  OPCS-4  OPCS-4  OPCS-4  OPCS-4  OPCS-4  OPCS-4  OPCS-4  OPCS-4  OPCS-4  OPCS-4  OPCS-4  OPCS-4  OPCS-4  OPCS-4  OPCS-4  OPCS-4  OPCS-4  OPCS-4 | K40  K41  K42  K43  K44.0  K44.1  K44.2  K44.8  K44.9  K45.0  K45.1  K45.2  K45.3  K45.4  K45.5  K45.6  K45.8  K45.9  K46.0  K46.1  K46.2  K46.4  K46.5  K46.8  K46.9 | CABG  (CALIBER phenotype) |
| SMR01 & SMR04 | ICD-10  ICD-10  ICD-10  ICD-10  ICD-10  ICD-10  ICD-10  ICD-10 | I20  I21  I22  I23  I24  I25  Z95.1  Z95.5 | CAD  (CALIBER phenotype) |
| SMR01 & SMR04 | OPCS-4  OPCS-4  OPCS-4  OPCS-4  OPCS-4  OPCS-4  OPCS-4  OPCS-4  OPCS-4  OPCS-4  OPCS-4  OPCS-4  OPCS-4  OPCS-4  OPCS-4  OPCS-4  OPCS-4  OPCS-4  OPCS-4  OPCS-4  OPCS-4  OPCS-4  OPCS-4  ICD-10  ICD-10  ICD-10  ICD-10  ICD-10  ICD-10  ICD-10  ICD-10  OPCS-4  ICD-10  ICD-10  ICD-10  ICD-10  ICD-10  ICD-10  ICD-10  ICD-10  ICD-10  ICD-10  ICD-10  ICD-10 | L29.0  L29.1  L29.2  L29.3  L29.4  L29.5  L29.6  L29.7  L29.8  L29.9  L30.0  L30.1  L30.3  L30.8  L30.9  L31.0  L31.1  L31.3  L31.4  L31.8  L31.9  L35.3  L37.2  I60  I61  I62.0  I62.1  I62.9  I69.0  I69.3  I63  U543  G46.3  G46.4  G46.5  G46.6  G46.7  I64  I67.2  I67.9  I69.1  I69.2  I69.4  I69.8 | Stroke  At least on record of  (haemorrhagic stroke, ischaemic stroke, or not otherwise specified stroke) based on CALIBER phenotypes. |
| SMR01 & SMR04 | ICD-10  ICD-10 | I42.1  I42.2 | HCM  (CALIBER phenotype) |
| SMR01 & SMR04 | OPCS-4  OPCS-4  OPCS-4  OPCS-4  OPCS-4  OPCS-4  OPCS-4  OPCS-4  OPCS-4  OPCS-4  OPCS-4  OPCS-4  OPCS-4  OPCS-4  OPCS-4  OPCS-4 | K59.1  K59.2  K59.3  K59.4  K59.5  K59.6  K59.7  K59.8  K59.9  K72.1  K72.2  K72.3  K72.4  K72.8  K72.9  X50.5 | ICD  (CALIBER phenotype) |
| SMR01 & SMR04 | OPCS-4  OPCS-4  ICD-10  ICD-10  ICD-10  ICD-10  ICD-10  ICD-10 | K50.2  K50.3  I20  I21  I22  I23  I24  I25 | IHD  (CALIBER phenotype) |
| SMR01 & SMR04 | OPCS-4  OPCS-4  OPCS-4  OPCS-4  OPCS-4  OPCS-4  OPCS-4  OPCS-4  OPCS-4  OPCS-4  OPCS-4  OPCS-4  OPCS-4  OPCS-4  OPCS-4  OPCS-4  OPCS-4  OPCS-4  OPCS-4  OPCS-4  OPCS-4  OPCS-4  OPCS-4  OPCS-4  OPCS-4  OPCS-4  OPCS-4  OPCS-4 | K60.1  K60.2  K60.3  K60.4  K60.5  K60.6  K60.7  K60.8  K60.9  K61.1  K61.2  K61.3  K61.4  K61.5  K61.6  K61.7  K61.8  K61.9  K73.1  K73.2  K73.3  K73.8  K73.9  K74.1  K74.2  K74.3  K74.8  K74.9 | Pacemaker  (CALIBER phenotype) |
| SMR01 & SMR04 | OPCS-4  OPCS-4  OPCS-4  OPCS-4  OPCS-4  OPCS-4  OPCS-4  OPCS-4  OPCS-4  OPCS-4  OPCS-4  OPCS-4  OPCS-4  OPCS-4  OPCS-4  OPCS-4  OPCS-4  OPCS-4  OPCS-4  OPCS-4  OPCS-4  OPCS-4  OPCS-4  OPCS-4  OPCS-4  OPCS-4  OPCS-4  OPCS-4  OPCS-4  OPCS-4  OPCS-4  OPCS-4  OPCS-4  ICD-10  ICD-10  ICD-10  ICD-10  ICD-10  ICD-10 | L50  L51  L52  L53.0  L53.1  L53.2  L53.8  L53.9  L54.0  L54.1  L54.2  L54.4  L54.8  L54.9  L58  L59  L60  L62.0  L62.1  L62.2  L62.8  L62.9  L63.0  L63.1  L63.2  L63.3  L63.5  L63.8  L63.9  L65.0  L65.1  L65.2  L65.3  I73.1  I73.8  I73.9  I74.3  I74.4  I74.5 | PAD  (CALIBER phenotype) |
| SMR01 & SMR04 | OPCS-4  OPCS-4  OPCS-4  OPCS-4  OPCS-4  OPCS-4  OPCS-4  OPCS-4 | K47.1  K49  K50.0  K50.1  K50.4  K50.8  K50.9  K75 | PCI  (CALIBER phenotype) |
| SMR01 & SMR04 | ICD-10  ICD-10  ICD-10  ICD-10  ICD-10  ICD-10  ICD-10  ICD-10  ICD-10  ICD-10  ICD-10  ICD-10  ICD-10  ICD-10  ICD-10  ICD-10  ICD-10  ICD-10  ICD-10  ICD-10  ICD-10  ICD-10  ICD-10  ICD-10 | I05  I06  I07  I08  I34  I35  I36  I37  Q22.1  Q22.2  Q22.3  Q22.4  Q22.5  Q22.8  Q22.9  Q23.0  Q23.1  Q23.2  Q23.3  Q23.8  Q23.9  Z95.2  Z95.3  Z95.4 | Valve Disease  (CALIBER phenotype) |
| **HF/ LD** | | | |
| SMR01 & SMR04 | ICD-10  ICD-10  ICD-10  ICD-10  ICD-10  ICD-10 | I13.0  I13.2  I11.0  I42.9  I42.0  I50 | HF  (Codes identified in previously published work) (2) |
| PIS | BNF | 020202  0202040D0  0202040B0  0202040T0  0202040U0  0202080D0  0202080C0  0202080K0 | Loop diuretics  Amiloride HCI with loop diuretics  Co-amilofruse (Amiloride hydrochloride/frusemide)  Spironolactone with loop diuretics  Triamterene with loop diuretics  Bumetanide/Amiloride hydrochloride  Bumetanide/potassium  Furosemide/potassium |
| **Cancer** | | | |
| SMR01 & SMR04 | \| ICD-10 \| \| --- \| | C00-C97 | Cancer |

SMR01, Scottish Morbidity Records for Acute Inpatient and Day Cases; SMR04, Mental Health Inpatient and Day Cases; PIS, Prescribing Information System for community-based prescriptions; AAA, Abdominal aortic aneurysm; AF, Atrial fibrillation; AFL, Atrial flutter; CABG; Coronary artery bypass graft; CAD, Coronary artery disease; COPD, Chronic obstructive pulmonary disease; CV, Cardiovascular; DM, Diabetes mellitus; HF, Heart failure; HCM, Hypertrophic cardiomyopathy; ICD, Implantable cardioverter defibrillator; IHD, Ischaemic heart disease; LD, Loop diuretics; MI, Myocardial infarction; PAD, Peripheral artery disease; PCI, Percutaneous coronary intervention.

**Supplemental Table 2: Codes used to identify hematological malignancies.**

| **Data Source** | **Code Source** | **Code** | **Description** |
| --- | --- | --- | --- |
| SMR01 & SMR04 | ICD-10 | C81 | Hodgkin lymphoma |
|  |  | C82 | Non-Hodgkin lymphoma |
|  |  | C83 | Non-Hodgkin lymphoma |
|  |  | C85 | Non-Hodgkin lymphoma |
|  |  | C86 | Non-Hodgkin lymphoma |
|  |  | C88.4 | Non-Hodgkin lymphoma |
|  |  | C90 | Myeloma |
|  |  | C91.0 | Acute lymphoblastic leukaemia |
|  |  | C91.1 | Chronic lymphoblastic leukaemia |
|  |  | C91.2 | Lymphoblastic leukaemia |
|  |  | C91.3 | Acute lymphoblastic leukaemia |
|  |  | C91.4 | Lymphoblastic leukaemia |
|  |  | C91.7 | Lymphoblastic leukaemia |
|  |  | C91.8 | Lymphoblastic leukaemia |
|  |  | C91.9 | Lymphoblastic leukaemia |
|  |  | C92.0 | Acute myeloid leukaemia |
|  |  | C92.1 | Chronic myeloid leukaemia |
|  |  | C92.2 | Chronic myeloid leukaemia |
|  |  | C92.5 | Acute myeloid leukaemia |
|  |  | C92.6 | Acute myeloid leukaemia |
|  |  | C92.7 | Myeloid leukaemia |
|  |  | C92.8 | Acute myeloid leukaemia |
|  |  | C92.9 | Myeloid leukaemia |
|  |  | C93.0 | Acute monocytic leukaemia |
|  |  | C93.1 | Chronic monocytic leukaemia |
|  |  | C93.7 | Monocytic leukaemia |
|  |  | C93.9 | Monocytic leukaemia |

SMR01, Scottish Morbidity Records for Acute Inpatient and Day Cases; SMR04, Mental Health Inpatient and Day Cases

**Supplemental Table 3: BNF codes used to identify dispensed medications at baseline.** Unless stated otherwise, all nested codes were included.

| **Medication Category** | **BNF Selection Code** | **Description** |
| --- | --- | --- |
| **Cardiovascular Medications** | | |
| ACEi | 0205051 | Angiotensin converting enzyme |
| ARB | 0205052  0206020Z0 | Angiotensin receptor blockers  Valsartan/Amlodipine |
| ARNI | 0205052AE | Sacubitril/Valsartan |
| Antiplatelet | 0209 | Antiplatelet |
| BB | 0204 | Beta-adrenergic blocking agents |
| Loop diuretics | 020202  0202040D0  0202040B0  0202040T0  0202040U0  0202080D0  0202080C0  0202080K0 | Loop diuretics  Amiloride HCI with loop diuretics  Co-amilofruse (Amiloride hydrochloride/frusemide)  Spironolactone with loop diuretics  Triamterene with loop diuretics  Bumetanide/Amiloride hydrochloride  Bumetanide/potassium  Furosemide/potassium |
| MRA | 0202030X0  0202030S0  0202040G0  0202040T0  0202040S0 | Eplerenone  Spironolactone  Co-flumactone(Hydroflumethiazide/spironolactone)  Spironolactone with loop diuretics  Spironolactone with thiazides |
| Oral anticoagulants | 020802 | Oral anticoagulants |
| Thiazides and related | 020201  0202040H  0202040C0  0204000Y0  020400040  0205051AB  0202040A0  0202040S0  0202040V0  0202080B0  0205052Y0  020400010  020400030  0204000F0  0204000Q0  0204000W0  0205051H0  0205051K0  0205051N0  0205051P0  0205051Z0  0205052A0  0205052P0  0205052R0  0205052X0  0205052AC  0205051G0 | Thiazides and related  Co-triamterzide (Triamterene/hydrochlorothiazide)  Co-amilozide(Amiloride hydrochloride/hydrochlorothiazide)  Co-prenozide (Oxprenolol hydrochloride/ cyclopenthiazide)  Co-tenidone (Atenolol/chlortalidone)  Perindopril tosilate/indapamide  Amiloride hydrochloride with thiazides  Spironolactone with thiazides  Triamterene with thiazides  Bendroflumethiazide/potassium  Olmesartan medoxomil/hydrochlorothiazide  Pindolol with diuretic  Timolol with diuretic  Atenolol with diuretic  Propranolol hydrochloride with diuretic  Metoprolol tartrate with diuretic  Enalapril maleate with diuretic  Lisinopril with diuretic  Perindopril erbumine with diuretic  Quinapril hydrochloride with diuretic  Perindopril arginine with diuretic  Irbesartan with diuretic  Losartan potassium with diuretic  Telmisartan with diuretic  Valsartan with diuretic  Olmesartan medoxomil/amlodipine/ hydrochlorothiazide  Co-zidocapt (Hydrochlorothiazide/captopril) |
| **Anti-Diabetic Medications** | | |
| Insulin | 0601023AU  060101 | Insulin/liraglutide  Insulin |
| Other hypoglycemics | 0601023AU  0601023S0  0601023V0  0601023B0  0601023W0  0601023S0  0601023V0  0601023B0  0601023W0  0601022B0  0601023AD  0601023AF  0601023AH  0601023AJ  0601023AL  0601023AP  0601023AR  0601023V0  0601023W0  0601023Z0  0601023AB  0601023AS  0601023Y0  0601023AI  0601023AQ  0601023AK  0601021A0  0601021M0  0601021X0  0601021P0  0601023AE  0601023AA | Insulin/liraglutide  Rosiglitazone  Metformin hydrochloride/ Rosiglitazone  Pioglitazone hydrochloride  Metformin hydrochloride/ Pioglitazone  Rosiglitazone  Metformin hydrochloride/ Rosiglitazone  Pioglitazone hydrochloride  Metformin hydrochloride/ Pioglitazone  Metformin  Metformin/sitagliptin  Linagliptin/metformin  Saxagliptin/metformin  Alogliptin/metformin  Dapagliflozin/metformin  Canagliflozin/metformin  Empagliflozin/metformin  Metformin/rosiglitasone  Metformin/pioglitasone  Metformin/vildagliptin  Liraglutide  Albiglutide  Exenatide  Lixisenatide  Dulaglitide  Alogliptin  Glimepiride  Glicazide  Tolbutamide  Glipizide  linagliptin  vildagliptin |
| **Others** | | |
| PPI | 010305 | PPI |
| Statins | 0212000B0  0212000AJ  0212000M0  0212000X0  0212000AA  0212000Y0  0212000AC | Atorvastatin  Fenofibrate/simvastatin  Fluvastatin sodium  Pravastatin sodium  Rosuvastatin calcium  Simvastatin  Simvastatin/ezetimibe |
| Corticosteroids (Inhaled) | 0302 | Corticosteroids (Inhaled) |
| Bronchodilators | 0301011R0  0301011F0  0301011V0  0301011B0  0301011E0  0301011X0  0301011Z0  0301011U0 | Salbutamol  Fenoterol  Terbutaline  Bambuterol  Formoterol  Indacterol  Olodaterol  Salmeterol |

BNF, British National Formulary; ACEi, Angiotensin-converting enzyme inhibitors; ARB, Angiotensin II receptor blocker; ARNI, Angiotensin receptor-neprilysin inhibitor; BB, Beta-blocker; MRA, Mineralocorticoid receptor antagonists; PPI, Proton pump inhibitor.

**Supplemental Table 4: Codes used to identify cause of death.**

| **Causes of Death** | **Description** |
| --- | --- |
| Cardiovascular | ICD-10 Chapter IX' Diseases of the circulatory system' (code range I00-I99), except for those classified as an infection. |
| Cancer | ICD-10 C00-Cx |
| Infections | See Supplementary Table 4 |
| Injury | V01-Y89 (excluding X41-X42, X44-X45), U12.9 |
| Other | Any code not included in any of the above categories |

**Supplemental Table 5: Codes used to identify death due to infection.** Unless stated otherwise, all nested codes were included.

| **Infection Classification** | **ICD-10 Code** | **Code Description** |
| --- | --- | --- |
| Infectious disease | A00 - B99 | Certain infectious and parasitic disease |
| Other  respiratory infections | H65  H65.0  H65.1  H65.2  H65.3  H65.4  H66.0  H66.1  H66.2  J01  J02  J03  J04  J05  J06  J09  J10  J11  J12  J13  J14  J15  J16  J17  J18  J20  J21  J22 | Nonsuppurative otitis media  Acute serous otitis media  Other acute nonsuppurative otitis media  Chronic serous otitis media  Chronic mucoid otitis media  Other chronic nonsuppurative otitis media  Acute suppurative otitis media  Chronic tubotympanic suppurative otitis media  Chronic atticoantral suppurative otitis media  Acute sinusitis  Acute pharyngitis  Acute tonsillitis  Acute laryngitis and tracheitis  Acute obstructive laryngitis [croup] and epiglottitis  Acute upper respiratory infections of multiple and unspecified sites  Influenza due to identified zoonotic or pandemic influenza virus  Influenza due to identified seasonal influenza virus  Influenza, virus not identified  Viral pneumonia, not elsewhere classified  Pneumonia due to Streptococcus pneumoniae  Pneumonia due to Haemophilus influenzae  Bacterial pneumonia, not elsewhere classified  Pneumonia due to other infectious organisms, not elsewhere classified  Pneumonia in diseases classified elsewhere  Pneumonia, organism unspecified  Acute bronchitis  Acute bronchiolitis  Unspecified acute lower respiratory infection |
| Other infectious diseases | G00 | Bacterial meningitis, not elsewhere classified |
|  | G03 | Meningitis due to other and unspecified causes |
|  | G04 | Encephalitis, myelitis and encephalomyelitis |

**R packages**

Tidyverse (3), gt (4), gtsummary (5), survival (6), survminer (7), lubridate (8), broom (9), forcats (10), rms (11).

**
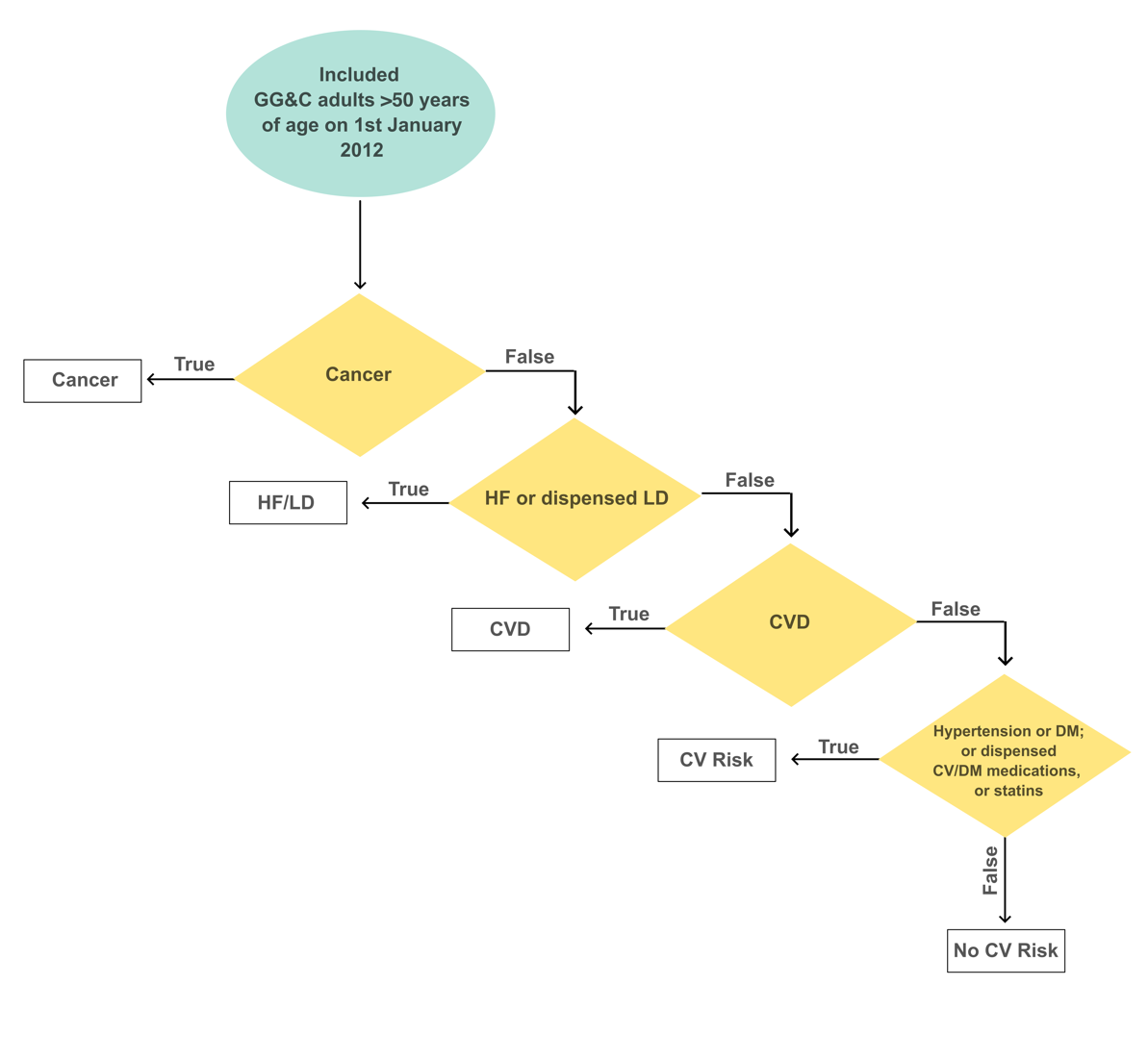
**

**Supplemental Figure 1: Flow diagram of categorization of study individuals based on prevalent disease and dispensed medications.** CV, Cardiovascular; CVD, Cardiovascular disease; DM, Diabetes mellitus; GG&C; Greater Glasgow & Clyde, HF, Heart failure; LD, Loop diuretic.

**Supplemental Table 6:** **Baseline demographics, comorbidities, blood tests and medications of the overall population and cardiovascular risk free, cardiovascular risk, cardiovascular disease, heart failure or loop diuretic and cancer cohorts with available NLR during 2014-2015.**

| **Characteristic** | **Overall** | **No CV Risk** | **CV Risk** | **CVD** | **HF/LD** | **Cancer** |
| --- | --- | --- | --- | --- | --- | --- |
| n | 223,388 | 70,239 | 106,973 | 14,490 | 23,009 | 8,677 |
| Age, years | 68  (60-77) | 62  (57-70) | 69  (62-77) | 73  (64-81) | 77  (68-84) | 71  (64-79) |
| Women | 125,493  (56%) | 41,458 (59%) | 58,645 (55%) | 6,741 (47%) | 14,509 (63%) | 4,140 (48%) |
| DM | 27,880  (12%) | Excluded | 18,601 (17%) | 2,694 (19%) | 5,401 (23%) | 1,184 (14%) |
| IHD | 13,362 (6%) |  | Excluded | 7,808 (54%) | 4,638 (20%) | 916 (11%) |
| MI | 6,451 (3%) |  |  | 3,580 (25%) | 2,463 (11%) | 408 (5%) |
| AF/AFL | 8,094 (4%) |  |  | 3,918 (27%) | 3,591 (16%) | 585 (7%) |
| PCI | 2,377 (1%) |  |  | 1,559 (11%) | 718 (3%) | 100 (1%) |
| CABG | 542 (<1%) |  |  | 284 (2%) | 237 (1%) | 21 (<1%) |
| Stroke | 4,905 (2%) |  |  | 3,440 (24%) | 1,135 (5%) | 330 (4%) |
| HF | 4,331(2%) |  |  | Excluded | 4,007 (17%) | 324 (4%) |
| COPD | 10,390 (5%) | 1,486 (2%) | 2,702 (3%) | 2,369 (16%) | 2,905 (13%) | 928 (11%) |
| Blood tests^a^ |  |  |  |  |  |  |
| Neutrophil count | 4.2  (3.3-5.4) | 3.9  (3.0-5.0) | 4.3  (3.4-5.4) | 4.5  (3.6-5.8) | 4.7  (3.7-6.0) | 4.4  (3.4-5.8) |
| Lymphocyte count | 1.9  (1.5-2.4) | 1.9  (1.5-2.4) | 1.9  (1.5-2.5) | 1.8  (1.4-2.4) | 1.8  (1.3-2.3) | 1.6  (1.2-2.1) |
| NLR^b^ | 2.2  (1.6-3.1) | 2.0  (1.5-2.7) | 2.2  (1.6-3.0) | 2.4  (1.8-3.4) | 2.7  (1.9-3.9) | 2.7  (1.9-4.1) |
| Available records of eGFR | 208,463  (93%) | 62,648  (89.2%) | 102,396  (95.7%) | 13,637  (94.3%) | 22,245  (96.5%) | 7,537  (95.7%) |
| eGFR^c^ | 82  (68-92) | 87 (78-95) | 81 (67-91) | 77 (63-88) | 65 (47-81) | 80 (64-90) |
| Available records of CRP | 73,306  (33%) | 26,066  (37.1%) | 31,270  (29.2%) | 4,879  (33.7%) | 8,006  (34.7%) | 3,085  (39.2%) |
| CRP | 0.3  (0.3-0.9) | 0.3 (0.2-0.7) | 0.3 (0.3-0.9) | 0.4 (0.3-1.1) | 0.6 (0.3-1.6) | 0.6 (0.3-2.0) |
| Available records of hemoglobin | 220,467  (99%) | 68,436  (97%) | 106,897  (100%) | 14,303  (99%) | 23,013  (100%) | 7,818  (99%) |
| Hemoglobin (Women) | 13.3  (12.4-14.1) | 13.5  (12.8-14.2) | 13.3  (12.5-14.2) | 13.0  (12.0-13.9) | 12.8  (11.7-13.8) | 12.8  (11.7-13.7) |
| Hemoglobin (Men) | 14.6  (13.5-15.5) | 14.9  (14.1-15.7) | 14.6  (13.6-15.5) | 14.2  (13.0-15.2) | 13.6  (12.3-14.8) | 13.6  (12.3-14.8) |
| Medications^a,d^ |  |  |  |  |  |  |
| Loop diuretics | 22,925 (10%) | Excluded | Excluded | Excluded | 21,804 (95%) | 1,121 (13%) |
| Thiazides and related | 33,891 (15%) |  | 29,442 (28%) | 1,940 (13%) | 1,323 (6%) | 1,186 (14%) |
| ACEi/ARBs | 77,423 (35%) |  | 54,187 (51%) | 7,139 (49%) | 13,259 (58%) | 2,838 (33%) |
| Beta-blocker | 52,876 (24%) |  | 32,585 (30%) | 7,091 (49%) | 11,017 (48%) | 2,183 (25%) |
| MRA | 2,860 (1%) |  | 677 (<1%) | 149 (1%) | 1,912 (8%) | 122 (1%) |
| Antiplatelet | 67,653 (30%) |  | 42,755 (40%) | 9,931 (69%) | 12,125 (53%) | 2,842 (33%) |
| Statins | 96,276 (43%) |  | 66,865 (63%) | 10,513 (73%) | 15,201 (66%) | 3,697 (43%) |
| Oral anticoagulants | 11,685 (5%) |  | 4,182 (4%) | 2,192 (15%) | 4,765 (21%) | 546 (6%) |
| Insulin | 4,859 (2%) |  | 2,816 (3%) | 474 (3%) | 13,52 (6%) | 217 (3%) |
| Other hypoglycemics | 23,869 (11%) |  | 16,755 (16%) | 1,927 (13%) | 4,274 (19%) | 913 (11%) |
| PPI | 90,524 (41%) | 20,282 (29%) | 45,574 (43%) | 7,374 (51%) | 12,997 (56%) | 4,297 (50%) |
| Corticosteroids (Inhaled) | 26,861 (12%) | 6,668 (10%) | 12,530 (12%) | 2,027 (14%) | 4,517 (20%) | 1,119 (13%) |
| Bronchodilators | 36,001 (16%) | 8,590 (12%) | 16,870 (16%) | 2,816 (19%) | 6,149 (27%) | 1,576 (18%) |

Median (IQR) for continuous variables; n (%) for categorical variables.

SI conversion factor: To convert C-reactive protein from mg/dL to mg/L, or haemoglobin from g/dL to g/L, multiply by 10.

ACEi, Angiotensin-converting-enzyme inhibitor; ARB, Angiotensin receptor blocker; AF, Atrial fibrillation; AFL, Atrial flutter; CABG, Coronary artery bypass graft; COPD, Chronic obstructive pulmonary disease; CV, Cardiovascular; CVD, Cardiovascular disease; DM, Diabetes mellitus; HF, Heart failure; IHD, Ischemic heart disease; LD, Loop diuretic; MI, Myocardial infarction; MRA, Mineralocorticoid receptor antagonist; NLR, Neutrophil-to-lymphocyte ratio; PCI, Percutaneous coronary intervention; PPI, Proton pump inhibitor.

^a^Most recent blood tests and medication use in the last 6 months before the index date.

Neutrophil and lymphocyte counts are in 10^3^/μL; CRP, C-reactive protein in mg/dL; Hemoglobin in g/dL.

^b^Calculated by dividing neutrophil count by lymphocyte count.

^c^eGFR, estimated glomerular filtration rate in ml/min/1.73m^2^ using CKD-EPI equation.

^d^Solely or in combination.

**Supplemental Table 7:** **Baseline demographics, comorbidities, blood tests and medications of the overall population and cardiovascular risk free, cardiovascular risk, cardiovascular disease, heart failure or loop diuretic and cancer cohorts with no available NLR during 2014-2015.**

| **Characteristic** | **Overall** | **No CV Risk** | **CV Risk** | **CVD** | **HF/LD** | **Cancer** |
| --- | --- | --- | --- | --- | --- | --- |
| n | 95,173 | 55,368 | 34,601 | 1,694 | 2,516 | 994 |
| Age, years | 62  (57-69) | 60  (56-66) | 66  (59-73) | 69  (61-79) | 74  (65-83) | 68  (62-76) |
| Women | 49,084 (52%) | 29,287 (53%) | 16,956 (49%) | 641 (38%) | 1,638 (65%) | 562 (57%) |
| DM | 4,261 (5%) | Excluded | 3,599 (10%) | 197 (12%) | 388 (15%) | 77 (8%) |
| IHD | 1,110 (1%) |  | Excluded | 808 (48%) | 247 (10%) | 55 (6%) |
| MI | 552 (<1%) |  |  | 386 (23%) | 143 (6%) | 23 (2%) |
| AF/AFL | 695 (<1%) |  |  | 436 (26%) | 212 (8%) | 47 (5%) |
| PCI | 265 (<1%) |  |  | 205 (12%) | 53 (2%) | 7 (<1%) |
| CABG | 73 (<1%) |  |  | 55 (3%) | 16 (<1%) | <5 (<1%) |
| Stroke | 506 (<1%) |  |  | 422 (25%) | 61 (2%) | 23 (2%) |
| HF | 303 (<1%) |  |  |  | 284 (11%) | 19 (2%) |
| COPD | 810 (<1%) | 252 (<1%) | 212 (<1%) | 148 (9%) | 117 (5%) | 81 (8%) |
| Blood tests^a^ |  |  |  |  |  |  |
| Available records of eGFR | 23,887  (25%) | 6,768  (12%) | 14,599  (42%) | 816  (48%) | 1237  (49%) | 467  (48%) |
| eGFR^c^ | 86 (75-94) | 90 (81-97) | 85 (75-93) | 85 (71-92) | 73 (57-86) | 86 (74-93) |
| Available records of CRP | 4,565  (5%) | 2,223  (4%) | 1,765  (5%) | 184  (11%) | 245  (9.7%) | 148  (15.2%) |
| CRP | 0.3 (0.3-0.6) | 0.3 (0.3-0.5) | 0.3 (0.3-0.6) | 0.4 (0.3-1.1) | 0.5 (0.3-1.2) | 0.6 (0.3-3.3) |
| Available records of hemoglobin | 13,668  (14%) | 6,047  (11%) | 6,080  (18%) | 465  (27%) | 714  (28%) | 362  (36%) |
| Hemoglobin (Women) | 13.5  (12.7-14.2) | 13.6  (12.9-14.2) | 13.5  (12.7-14.2) | 13.0  (12.1-13.8) | 12.9  (11.9-14.0) | 13.0  (11.6-13.8) |
| Hemoglobin (Men) | 14.8  (14.0-15.6) | 14.9  (14.2-15.7) | 14.8  (14.0-15.6) | 14.4  (13.4-15.4) | 13.8  (13.0-15.0) | 14.0  (12.1-15.0) |
| Medications^a,d^ |  |  |  |  |  |  |
| Loop diuretics | 2,451 (3%) | Excluded | Excluded | Excluded | 2384 (95%) | 67 (7%) |
| Thiazides and related | 10,664 (11%) |  | 10,235 (30%) | 200 (12%) | 118 (5%) | 111 (11%) |
| ACEi/ARBs | 18,776 (20%) |  | 16,509 (48%) | 774 (46%) | 1,259 (50%) | 234 (24%) |
| Beta-blocker | 12,078 (13%) |  | 10,098 (29%) | 804 (47%) | 1,021 (41%) | 155 (16%) |
| MRA | 201 (<1%) |  | 95 (<1%) | 8 (<1%) | 91 (4%) | 7 (<1%) |
| Antiplatelet | 12,123 (13%) |  | 9,708 (28%) | 1,095 (65%) | 1,119 (44%) | 201 (20%) |
| Statins | 21,915 (23%) |  | 19,210 (56%) | 1,091 (64%) | 1,344 (53%) | 270 (27%) |
| Oral anticoagulants | 1,456 (2%) |  | 869 (3%) | 203 (12%) | 355 (14%) | 29 (3%) |
| Insulin | 696 (1%) |  | 584 (2%) | 26 (2%) | 75 (3%) | 11 (1%) |
| Other hypoglycemics | 3,720 (4%) |  | 3,220 (9%) | 129 (8%) | 320 (13%) | 51 (5%) |
| PPI | 18,118 (19%) | 7,724 (14%) | 8,448 (24%) | 605 (36%) | 1,039 (41%) | 302 (30%) |
| Corticosteroids (Inhaled) | 6,168 (7%) | 3,129 (6%) | 2,461 (7%) | 150 (9%) | 340 (14%) | 88 (9%) |
| Bronchodilators | 7,654 (8%) | 3,754 (7%) | 3,137 (9%) | 195 (12%) | 440 (17%) | 128 (13%) |

Median (IQR) for continuous variables; n (%) for categorical variables.

SI conversion factor: To convert C-reactive protein from mg/dL to mg/L, or haemoglobin from g/dL to g/L, multiply by 10.

ACEi, Angiotensin-converting-enzyme inhibitor; ARB, Angiotensin receptor blocker; AF, Atrial fibrillation; AFL, Atrial flutter; CABG, Coronary artery bypass graft; COPD, Chronic obstructive pulmonary disease; CV, Cardiovascular; CVD, Cardiovascular disease; DM, Diabetes mellitus; HF, Heart failure; IHD, Ischemic heart disease; LD, Loop diuretic; MI, Myocardial infarction; MRA, Mineralocorticoid receptor antagonist; NLR, Neutrophil-to-lymphocyte ratio; PCI, Percutaneous coronary intervention; PPI, Proton pump inhibitor.

^a^Most recent blood tests and medication use in the last 6 months before the index date.

Neutrophil and lymphocyte counts are in 10^3^/μL; CRP, C-reactive protein in mg/dL; Hemoglobin in g/dL.

^b^Calculated by dividing neutrophil count by lymphocyte count.

^c^eGFR, estimated glomerular filtration rate in ml/min/1.73m^2^ using CKD-EPI equation.

^d^Solely or in combination.


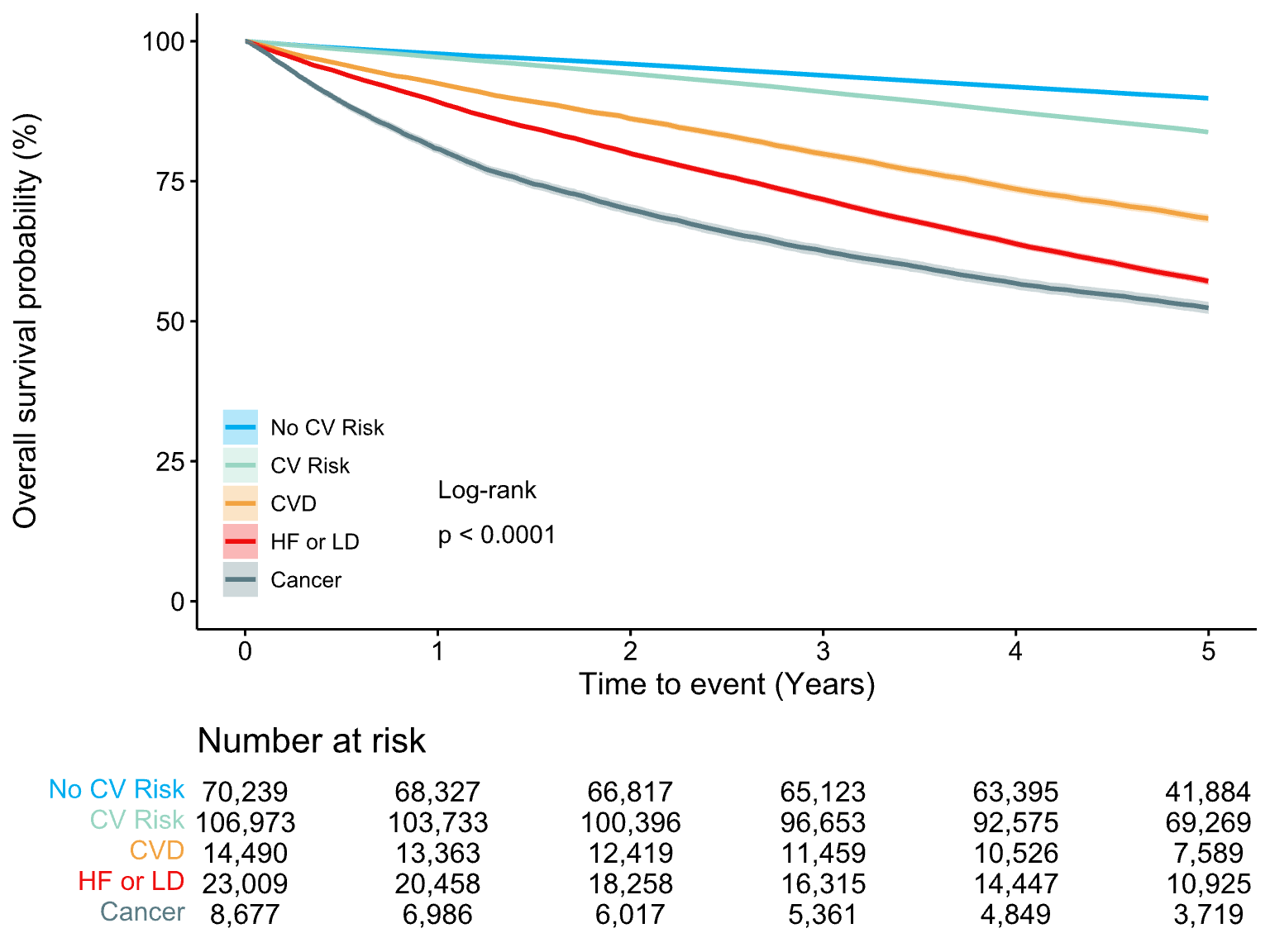


**Supplemental Figure 2: Kaplan-Meier estimates of 5-year survival between groups by prevalent cardiovascular risk factors/disease or cancer.** CV, Cardiovascular; CVD, Cardiovascular disease; HF, Heart failure; LD, Loop diuretic

**
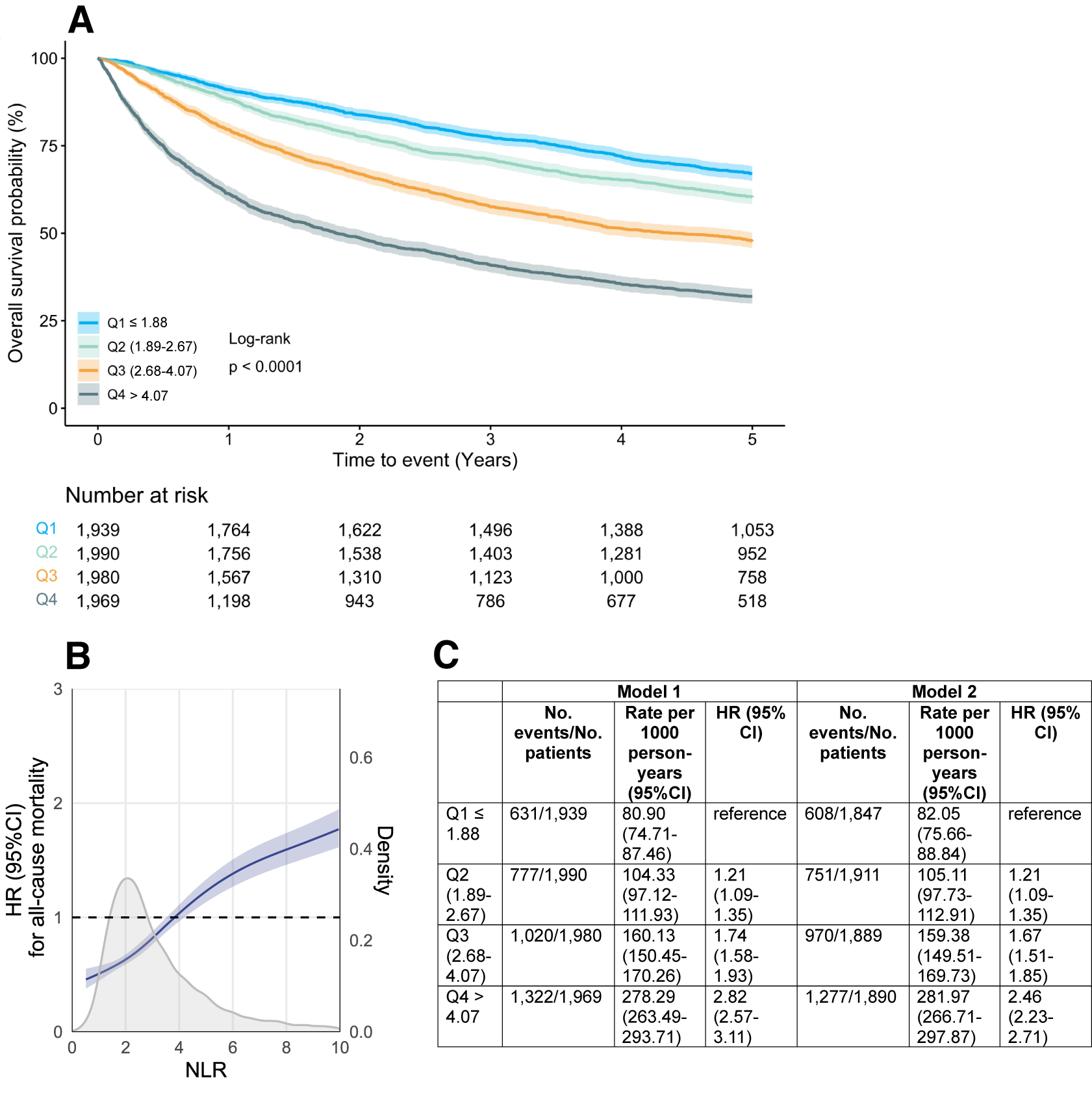
**

**Supplemental Figure 3: 5-year survival analysis in people with prevalent cancer excluding those with records of hematological cancer.** (A) Kaplan-Meier estimates by quartiles of NLR. (B) Association between NLR and all-cause mortality, adjusting for age, sex, eGFR and hemoglobin. (C) Association between NLR quartiles and all-cause mortality taking the 1^st^ quartile as reference. Model 1 adjusted for age and sex. Model 2 adjusted for age, sex, eGFR and hemoglobin.


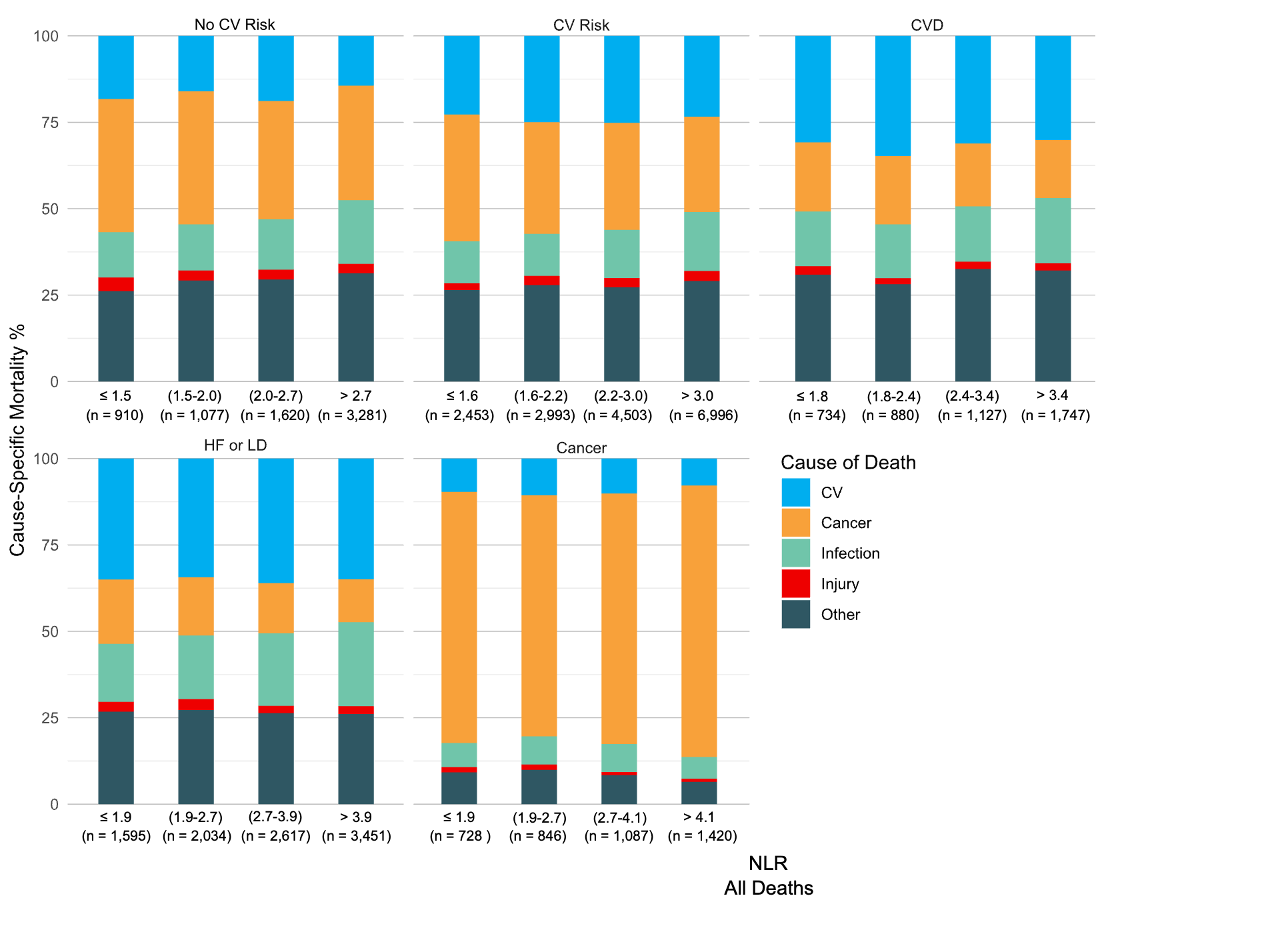


**Supplemental Figure 4: Cause specific mortality by quartiles of baseline neutrophil-to-lymphocyte ratio (NLR).** CV, Cardiovascular; CVD, Cardiovascular disease; HF, Heart failure; LD, Loop diuretic.

# **References**

1. Alvarez-Madrazo S, McTaggart S, Nangle C, Nicholson E, Bennie M**.** Data Resource Profile: The Scottish National Prescribing Information System (PIS). Int J Epidemiol. 2016;45(3):714-5f.

2. Conrad N, Judge A, Tran J, Mohseni H, Hedgecott D, Crespillo AP, et al. Temporal trends and patterns in heart failure incidence: a population-based study of 4 million individuals. Lancet. 2018;391(10120):572-80.

3. Wickham H, Averick M, Bryan J, Chang W, McGowan LDA, François R, et al. Welcome to the Tidyverse. Journal of open source software. 2019;4(43):1686.

4. Iannone R, Cheng J, Schloerke B, Hughes E**.** gt: Easily Create Presentation-Ready Display Tables. R package version 0.10.0 https://CRAN.R-project.org/package=gt ed; 2023.

5. Daniel SD, Karissa W, Michael C, Jessica LA, Joseph L**.** Reproducible Summary Tables with gtsummary Package. The R Journal. 2021;13(1):570-80.

6. Therneau TM**.** A Package for Survival Analysis in R. R package version 3.3-1 https://CRAN.R-project.org/package=survival ed; 2022.

7. Kassambara A, Kosinski M, Biecek P**.** survminer: Drawing Survival Curves using `ggplot2'. R package version 0.4.9 https://CRAN.R-project.org/package=survminer ed; 2021.

8. Grolemund G, Wickham H**.** Dates and Times Made Easy with lubridate. Journal of Statistical Software. 2011;40(3):1--25.

9. Robinson D, Hayes A, Couch S**.** broom: Convert Statistical Objects into Tidy Tibbles. R package version 0.7.10 https://CRAN.R-project.org/package=broom ed; 2021.

10. Wickham H**.** forcats: Tools for Working with Categorical Variables (Factors). R package version 0.5.1 https://CRAN.R-project.org/package=forcats ed; 2021.

11. Harrel Jr FE**.** rms: Regression Modeling Strategies. R package version 6.7-1 https://CRAN.R-project.org/package=rms ed; 2023.
